# *Prunus lusitanica* L. Fruits as a Novel Source of Bioactive Compounds with Antioxidant Potential: Exploring the Unknown

**DOI:** 10.3390/antiox11091738

**Published:** 2022-08-31

**Authors:** Ana Santos Abraão, Nelson Fernandes, Amélia M. Silva, Raúl Domínguez-Perles, Ana Barros

**Affiliations:** 1 Centre for the Research and Technology of Agro-Environmental and Biological Sciences, University of Trás-os-Montes and Alto Douro (CITAB)/Institute for Innovation, Capacity Building and Sustainability of Agri-Food Production (Inov4Agro), 5000-801 Vila Real, Portugal; 2 Department of Biology and Environment (DeBA-ECVA), University of Trás-os-Montes and Alto Douro (UTAD), 5001-801 Vila Real, Portugal; 3 Phytochemistry and Healthy Foods Lab (LabFAS), CEBAS-CSIC (Consejo Superior de Investigaciones Científicas), Campus Universitario de Espinardo, Edif. 25, 30100 Murcia, Spain

**Keywords:** *Prunus lusitanica* L. fruits, (poly)phenolic profile, antioxidant capacity, chromatography, mass spectrometry

## Abstract

*Prunus lusitanica* L., also known as Portuguese laurel or locally known as ‘azereiro’, is a rare species with ornamental and ecological value. Only two studies regarding the bioactivity and chemical composition of its leaves were reported to date. Thus, the present study aims to qualitatively and quantitatively evaluate the phenolic profile, through HPLC-PAD-ESI-MS/MS (high-performance liquid chromatography–photodiode array detection–electrospray ionization tandem mass spectrometry), as well as the radical scavenging capacity, through ABTS (2,2’-azino-bis-3-ethylbenzothiazoline-6-sulfonic acid) and DPPH (2,2-diphenyl-1 picrylhydrazyl), and the reducing power (FRAP, ferric reducing antioxidant power) assays, of P. lusitanica fruits during a 4-year study. In total, 28 compounds were identified and quantified in the fruits, including 21 hydroxycinnamic acids (60.3%); 2 flavan-3-ols (27.9%), 2 anthocyanins (10.5%), 2 flavonols (1.0%), and 1 secoiridoid (0.3%). High antioxidant capacity was observed, with ABTS values ranging from 7.88 to 10.69 mmol TE (Trolox equivalents)/100 g fw (fresh weight), DPPH values from 5.18 to 8.17 mmol TE/100 g fw, and FRAP values from 8.76 to 11.76 mmol TE/100 g fw. According to these results, it can be concluded that these are rich sources of phenolic compounds with very promising antioxidant capacity and, therefore, with potential applications in the food and/or phytopharmaceutical sectors.

## 1. Introduction

Reactive oxygen species (ROS) are generated in living organisms as a result of mitochondrial respiration [1]. The imbalance between the detoxifying cell capacity and the harmful effect of ROS triggers oxidative stress, which is critical in the development of several diseases [2,3]. In recent years, the increase in stress-mediated diseases has resulted in the need to identify more of the molecular tools cells are using to tackle the negative effects of ROS. Among other strategies, this challenge has been addressed by the use of powerful naturally occurring antioxidants, as in the case of (poly)phenols [4].

Phenolic compounds are secondary metabolites of plants that are responsible for the healthy properties of plant extracts and their broad use in galenic medicine [5]. The attention of the scientific community on these compounds has increased in the last decades because of their beneficial effects on health [6,7]. Indeed, the biological advantages have been associated with their ability to scavenge ROS by transferring electrons to free radicals and activating antioxidant enzymes [4], whose capacities help to reduce oxidative stress and the severity of inflammatory processes, contributing importantly to the prevention of human pathologies [7,8].

There are 400–430 species in the *Prunus* genus, which belongs to the Rosaceae family [9]. The species within this genus are spread all over the world, although only 98 species are of any value [10]. The economic importance of *Prunus* species is based mostly on their fruits, such as cherries, plums, peaches, apricots, and/or almonds, which are marketed and consumed globally, as well as their oils, timbers, and ornamentals [11,12]. Besides the nutritional features, the *Prunus* species have been highlighted as valuable sources of bioactive phytochemicals, which stresses the healthy attributions of these foodstuffs [9]. In this regard, over 500 listed bioactive compounds have been isolated from this single genus so far [13], with (poly)phenols being of special relevance [13,14,15,16,17,18,19,20]. Altogether, the *Prunus* species composition confers to them a wide range of medicinal uses [10].

The laurel cherry, *Prunus lusitanica* L., also known as cherry bay or Portuguese laurel, can adapt to its surroundings and help to maintain ecological balance and ecosystem sustainability [21]. Despite its unique qualities and relative importance, there are only two studies in the literature about the chemical composition of its leaf extracts in bioactive chemicals as well as their bioactivities [21,22]. Considering that uncommon fruit species are currently receiving a lot of attention and their health benefits are at the forefront, it is of utmost importance to unravel their phytochemical composition and antioxidant capacity, adding value to them and promoting their use [23]. At present, to the best of our knowledge, this species grows spontaneously in different places in Portugal in the continental climate and has not been included in any domestication program (except for ornamental purposes, https://www.ncbi.nlm.nih.gov/Taxonomy/Browser/wwwtax.cgi?mode=Info&id=194545, accessed on 17 August 2022). However, the identification of promising phytochemical properties could quickly boost the initiation of such a domestication process to take advantage of the phytochemical burden found in this *Prunus* species.

The aim of this study was to analyze the phenolic profile of *Prunus lusitanica* L. fruits (grown in northern Portugal) and correlate it with the antioxidant capacity during a 4-year study, focusing on its potential future applications in the food and/or phytopharmaceutical industries, taking into account the uses and applications of other related species belonging to the same genus. To the best of our knowledge, this is the first study that addresses both the phenolic composition of the fruits of this species as well as their antioxidant capacity.

## 2. Materials and Methods

### 2.1. Chemicals and Reagents

Sodium nitrate, aluminum chloride, and sodium hydroxide, all extra pure (>99%), and methanol (≥99.9%) were acquired from Merck (Merck, Darmstadt, Germany). Folin–Ciocalteu’s reagent, 3,4,5-trihydroxy benzoic acid (gallic acid, >99.0%), acetic acid (>99.0%), and sodium hydroxide (98.0%) were purchased from Panreac (Panreac Química S.L.U., Barcelona, Spain). Sodium molybdate (99.5%) was obtained from Chem-Lab (Chem-Lab N.V., Zedelgem, Belgium). The compounds 2,2-azino-bis (3-ethylbenzothiazoline-6-sulphonic acid) diammonium salt (ABTS^•+^, ≥98.0%), 2,2-diphenyl-1-picrylhidrazyl radical (DPPH, ≤100.0%), potassium phosphate (≥99.0%), catechin (98%), potassium persulfate (≥99.0%), sodium acetate (≥98.0%), 2,4,6-Tripyridyl-*s*-Triazine (TPTZ iron reagent, ≥98.0%), acetic acid (99.7%), hydrochloric acid (≥98.0%), and iron (III) chloride (≥99.9%) were obtained from Sigma-Aldrich (Sigma-Aldrich Produktions GmbH, Steinheim, Germany). Additionally, 6-hydroxy-2,5,7,8-tetramethylchroman-2-carboxylic acid (Trolox, ≥98.0%) was purchased from Fluka Chemika (Fluka Chemika, Neu-Ulm, Switzerland). The standards for chromatographic determinations were purchased from Sigma-Aldrich (St Louis, Steinheim, Germany). Methanol, acetonitrile, and acetic acid (LC-MS-grade solvents, purity ≥99.9%) were provided by J.T. Baker (Philipsburg, NJ, USA). Milli-Q purified water (Millipore, Bedford, MA, USA) was used for all the extraction and chromatographic analyses.

### 2.2. Plant Material

The sampling took place in three different locations at the Campus of the University of Trás-os-Montes and Alto Douro (UTAD), which is set in an ecocampus integrating one of Europe’s largest botanical gardens and is located in northern Portugal (Vila Real). The sampling of plant material was performed for four consecutive years (2016–2019) when the *Prunus lusitanica* L. fruits were fully matured according to the criteria of uniformity of the purple color throughout the bunch (early October). The meteorological data corresponding to the seasons 2016, 2017, 2018, and 2019 are presented in Table 1. The *Prunus lusitanica* specimens characterized in the present work were duly identified in the database of the University of Trás-os-Montes and Alto Douro’s botanical garden.

About 600 fruits were collected randomly from the trees located in three different locations within the botanical gardens of the UTAD ecocampus. Fruits were transported to the laboratory in freezer cabinets, where they were thoroughly mixed to be bulked into three well-mixed replicates. The whole fruits (pulp and stone) were frozen at −80 °C, and freeze-dried (VirTis Benchtop Pro Freeze-drier with Omnitronics^TM^, SP industries, Inc, Warminster, PA, USA). For further investigation, the freeze-dried replicates were ground into a fine powder and stored hermetically protected from light.

### 2.3. Preparation of Prunus lusitanica L. Fruit Extracts

To assess the phenolic content as well as the antioxidant capacity, three extracts of each sample were prepared sequentially by mixing 40 mg of powder with 1.5 mL of deionized water/ethanol (30:70, *v*/*v*) in 0.1% 32.6 M HCl. The mixture was properly homogenized and stirred in an orbital shaker (GFL 3005, GEMINI, Apeldoorn, The Netherlands) at room temperature for 30 min. Afterwards, the mixtures were centrifuged at 2291 g at 4 ℃ for 15 min (Sigma 2-16KL Refrigerated Centrifuges, Sigma Laborzentrifugen, Berlin, Germany), and the supernatants were collected. This process was repeated four times. The final extraction volume was made up to 10 mL with the extracting solvent using a volumetric flask and filtered through 0.2 μm regenerated cellulose filters (OlimPeak, Teknokroma, Barcelona, Spain).

### 2.4. Determination of the Antioxidant Capacity

Three different spectrophotometric methods were used to characterize the antioxidant capacity of the extracts: ABTS and DPPH radical scavenging methods and FRAP (ferric reducing antioxidant power). The DPPH- and ABTS-based determinations were performed as described by Lemos et al. [24], and FRAP was carried out as reported by Yu et al. [25]. Similar to what was performed in the quantification of the different phenolic classes, the three antioxidant assays were carried out on a microscale using 96-well microplates (PrimeSurface MS- 9096MZ, Frilabo, Maia, Portugal) and microplate readers (Multiskan GO Microplate Photometer, Thermo Fisher Scientific, Vantaa, Finland).

After a reaction period of 12–16 h, the ABTS^•+^ radicals were prepared through the combination of 5 mL of ABTS stock solution (7.0 mM in water) with 88 μL of potassium persulfate (148 mM) and diluted to a working solution with sodium acetate buffer (20 mM, pH 4.5) with an absorbance of 0.70 at 734 nm. Thereafter, 188 μL of ABTS working solution and 12 μL of each extract (70% hydro-ethanol used as a blank) were combined and left to react, protected from light. After 30 min, the absorbance was measured at 734 nm to determine the radical scavenging capacity.

The DPPH working solution was made by diluting the DPPH^•^ radical (8.87 mM in methanol) in a 70:30 *v*/*v* methanol/water solution until an absorbance of 1.00 at 520 nm. The radical scavenging activity was determined by the measurement of the absorbance at the same wavelength in a mixture of 190 μL of the DPPH working solution and 10 μL of the extract (70% hydro-ethanol used as a blank) (after a 30 min reaction, protected from light, at room temperature). In the cases of the DPPH and ABTS methods, the scavenging capacity of the samples was calculated by the interpolation of the Trolox calibration curve. The results were expressed in millimoles of Trolox equivalents per 100 g of fruit fresh weight (mmol TE/100 g fw).

To measure the ferric reducing antioxidant power (FRAP), extracts (20 μL) were placed in a microplate well, followed by 180 μL of FRAP working solution (composed of 1 volume of TPTZ (10 mM dissolved in hydrochloric acid), 1 volume of ferric chloride (20 mM in water), and 10 volumes of acetate buffer (300 mM, pH 3.6)). The mixture was incubated at 37 °C, protected from light, for 30 min. Afterwards, the absorbance was measured at 593 nm. Again, Trolox was used as a standard, and the results were expressed in mmol TE/100 g fw.

### 2.5. HPLC–PAD–ESI-MS/MS Analysis of the Quantitative (Poly)phenolic Profile of Prunus lusitanica L. Fruits

The identification and quantification of the phenolic compounds were performed according to Abellán et al. [26] with minor modifications. In some detail, chromatographic separations were carried out using a Kinetex Luna C18 column (250 × 4.6 mm, 2.6 µm particle size; Phenomenex, Macclesfield, UK) with a security guard C18-ODS cartridge system (Phenomenex). The chromatographic resolution of the phenolic profile was achieved using deionized water/formic acid (99:1, *v*/*v*) (A) and acetonitrile (B) as chromatographic solvents using the following gradient (Time, %B): (0, 5%), (30, 25%), (35, 50%), (37, 50%), and (38, 95%). The flow rate was 0.8 mL/min, and the injection volumes were 20 µL. The HPLC system was equipped with an Agilent 1100 Series diode array and a mass detector in series (Agilent Technologies, Waldbronn, Germany). It consisted of a G1312A binary pump, a G1313A autosampler, a G1322A degasser, and a G1315B photodiode array detector controlled by ChemStation software version 08.03 (Agilent Technologies, Waldbronn, Germany). Spectroscopic data from all peaks were accumulated in the range of 240–600 nm, and the spectral data were recorded at 280, 320, 330, and 520 nm.

The mass detector was a G2445A Ion-Trap Mass Spectrometer equipped with an electrospray ionization (ESI) system and controlled by LCMSD software version 4.1 (Agilent, Waldbronn, Germany). Nitrogen was used as a nebulizing gas at a pressure of 60 psi, and the flow was adjusted to 11 L/min. The heated capillary and voltage for ionization were maintained at 350 °C and 5 kV, respectively. Collision-induced fragmentation experiments were performed in the ion trap using helium as a collision gas, with voltage ramping cycles from 0.3 up to 2 V. The full scan mass covered the range from *m*/*z* 100 up to *m*/*z* 1600. Mass spectrometry data were acquired in the negative and positive ionization modes. Total ion chromatograms were recorded as two alternating automatic scan events: full scan mass spectra (MS) and MS/MS for the fragmentation of the most abundant molecular ions. The identification of the individual phenolic compounds was performed by analyzing the retention time (min), parent ions, and fragmentation patterns in comparison with authentic standards (3-*O*-caffeoylquinic acid, Caffeoyl di-hexoside, 3-*p*-coumaroylquinic acid, 4-*p*-coumaroylquinic acid, catechin, B-type proanthocyanidin trimer, quercetin-3-*O*-glucoside, quercetin-3-*O*-rutinoside, and cyanidin-3-*O*-glucoside) and, when they were not available, descriptions available in the literature. Phenolic compounds were quantified by PDA chromatograms recorded at the abovementioned wavelengths, using freshly prepared calibration curves of 3-*O*-caffeoylquinic acid for phenolic acids (*r*^2^ = 0.9995), swertiamarin for secoiridoids (*R*^2^ = 0.9969), catechin for flavan-3-ols (*r*^2^ = 0.9991), quercetin-3-*O*-glucoside for flavonols (*r*^2^ = 0.9999), and cyanidin-3-*O*-glucoside for anthocyanins (*r*^2^ = 0.9985) for each day of analysis.

### 2.6. Statistical Analysis

All the assays were carried out in triplicate (*n* = 3) for three different extracts, and the results are expressed as means with the indication of the least significant difference (LSD) value as a dispersion parameter. The statistical differences were obtained through a one-way analysis of variance (ANOVA) and a multiple range test (Tukey’s test) for a *p* < 0.05. A principal component analysis (PCA) and Pearson correlations were conducted in the MATLAB R2019b environment (MathWorks, Inc., Natick, MA, USA).

## 3. Results and Discussion

### 3.1. Polyphenolic Profile

The identification of the phenolic compounds present in *Prunus lusitanica* fruits was performed by HPLC–PAD–ESI-MS/MS analysis, and data of the retention time, λ_max_, pseudomolecular ion, and main fragment ions in MS^n^ in comparison with the literature are shown in Table 2. Accordingly, in this study 28 phenolic compounds were identified and quantified in *Prunus lusitanica* fruits. These compounds belong to different classes, namely, hydroxycinnamic acids, secoiridoids, flavan-3-ols, flavonols, and anthocyanins. The analyzed extracts gathered over four consecutive years in three different locations displayed a similar phenolic profile.

#### 3.1.1. Hydroxycinnamic Acids

Regarding phenolic acids, 21 different compounds were identified in *Prunus lusitanica* fruits, all of them belonging to the hydroxycinnamic acid derivative subgroup (compounds 1–**5**, **7**, **10**–**19**, and **22**–**26**).

**Peak 1** ([M−H]^−^ at *m*/*z* 353) was identified as 3-*O*-caffeoylquinic acid, yielding the base peak at *m*/*z* 191, corresponding to deprotonated quinic acid, [quinic acid−H]^−^, and another characteristic ion at *m*/*z* 179, [caffeic acid−H]^−^, in MS^2^ [27,28,29].

**Peak 2** presented a UV spectrum similar to caffeic acid, with a λ_max_ around 328 nm, and a pseudo molecular ion [M–H]^−^ at *m*/*z* 503. The MS fragmentation gave an *m*/*z* 341 ion (deprotonated caffeoyl-hexosides) (−162 arbitrary mass units(amu), loss of a hexosyl moiety) and *m*/*z* 179 [caffeic acid–H]^−^ (−162 amu, corresponding to the loss of a second hexosyl moiety), 161([caffeic acid–H–H_2_O]^−^), and 135 ([caffeic acid–H–CO_2_]^−^), typical of a caffeic acid structure. This compound was identified as a caffeoyl di-hexoside [30,31]. This type of compound has already been identified in fruits belonging to the *Prunus* genus, such as *Prunus spinosa* [15] and *Prunus avium* [18,32].

Two compounds detected in the extracted ion chromatogram produced a pseudomolecular ion at *m*/*z* 337. The first eluting isomer (Table 2), **peak 4**, yielded the MS^2^ base peak at *m*/*z* 163 ([*p*–coumaric acid–H]^−^), which is characteristic of 3-*p*-coumaroylquinic acid. The second eluting isomer, **peak 10**, produced the MS^2^ base peak at *m*/*z* 173 ([quinic acid–H_2_O–H]^−^), indicating quinic acid substitution at position 4, which is characteristic of 4-*p*-coumaroylquinic acid [27,33,34].

**Peak 5** gave a parent ion [M–H]^−^ at *m*/*z* 487 and displayed a fragmentation pattern characterized by fragments at *m*/*z* 341, corresponding to caffeic acid hexoside, *m*/*z* 179 [caffeic acid–H]^−^ (−162 amu, loss of a hexosyl moiety), and *m*/*z* 163 ([*p*–coumaric acid–H]^−^). Considering the fragmentation pattern observed and the literature, the compound represented by peak 5 was identified as caffeic acid-*O*-(coumaroyl)hexoside [35,36].

**Peak 7** was identified as caffeoyl-isocitrate based on the parent ion [M–H]^−^ at *m*/*z* 353, and the fragment ions at *m*/*z* 173 resulting from the elimination of the caffeic acid unit (loss of 180 amu) and *m*/*z* 111 and 155, which represents a distinctive feature of acyl-isocitrates [37,38]. This compound has already been identified in other berry fruits, namely, *Vaccinium cylindraceum* [39] and *Myrica faya* [40].

**Peaks 3**, **11**, **12**, **13–19**, and **22–26** were assigned as acetyl-*p*-coumaroylsucroses. **Peak 3** presented an [M−H]^−^ ion at *m*/*z* 487. Together with this, it was also possible to observe an ion at *m*/*z* 307, corresponding to the loss of a glucose molecule [M−H−180]^−^, and a *p*-coumaroyl residue at *m*/*z* 145 originated from the loss of a glucosyl residue. In this sense, the compound corresponding to **peak 3** was identified as *p*-coumaroyl-3-*O*-sucrose. **Peaks 11** and **12** presented an [M−H]^−^ ion at *m*/*z* 529 and fragment ions at *m*/*z* 487 (base peak), corresponding to the loss of an acetyl moiety [M−H−42]^−^ and *m*/*z* 349, which originated from the loss of a glucose moiety [M−H−180]^−^, suggesting the linking of the acetyl moiety to the internal glycone (fructosyl moiety). According to that, **peaks 11** and **12** were assigned as mono-*O*-acetyl-*p*-coumaroylsucroses. **Peaks 13**, **14**, **15**, and **18** were identified as isomers of di-*O*-acetyl-*p*-coumaroyl sucrose with identical [M−H]^−^ at *m*/*z* 571. The observation of the fragment ions [M−H−2 × 42]^−^ at *m*/*z* 487 and [M−H−180−2 × 42]^−^ at *m*/*z* 307 indicated that both acetyls were linked to glucose. **Peaks 16**, **17**, **19**, **22**, **23**, and **24** were assigned tri-*O*-acetyl-3-*O*-*p*-coumaroyl sucrose isomers with [M−H]^−^ at *m*/*z* 613. **Peaks 16**, **17**, **19**, **22**, and **24** presented ion [M−H−180−2×42]^−^ at *m*/*z* 349, indicating that one acetyl residue was linked to fructose while the other two acetyls were on glucose. In the case of **peak 23**, this fragment was not observed, indicating the possibility of the three acetyl groups being linked to glucose. Peaks 25 and 26 were identified as isomers of tetra-*O*-acetyl-*p*-coumaroylsucrose with [M−H]^−^ at *m*/*z* 655. **Peak 25** gave a fragment ion [M−H−180−3 × 42]^−^ at *m*/*z* 349, indicating that an acetyl group was linked to fructose. In the case of **peak 26**, this fragment was absent [41,42].

Although the fragmentation pattern was conclusive regarding the identification of the type of compounds belonging to the acetyl-*p*-coumaroylsucrose group, it was not sufficient to establish their positional isomerism.

#### 3.1.2. Secoiridoids

In the case of **peak 6**, given the scarcity of bibliographic references that address it and taking into account the only one found that addresses the mass spectrometry of this compound [43] by comparing the molecular ion at [M−H]^−^ at *m*/*z* 581 as well as the fragmentation pattern, this compound was tentatively identified as 6’-*O*-*β*-D-glucosyl swertiamarin.

#### 3.1.3. Flavan-3-ols

**Peak 8** gave a parent ion [M−H]^−^ at *m*/*z* 289. The fragment at *m*/*z* 245 results from the loss of C_2_H_4_O group. The elimination of the B ring after the heterocyclic ring fission originated with the fragment ion at *m*/*z* 165. The loss of the catechol group led to the formation of the fragment at *m*/*z* 179. Thus, **peak 8** was assigned as catechin [16,44].

Regarding **Peak 9**, it displayed a parent ion [M−H]^−^ at *m*/*z* 865, with a base peak at *m*/*z* 695 formed as a result of the loss of a retro-Diels–Alder (RDA) fragment (−152 amu) and a water molecule (−18 amu). Following quinone methide (QM) cleavage of the interflavan bond, the sequence was confirmed by the observation of the fragment ion at *m*/*z* 287, which was derived from the extension unit, in addition to peaks *m*/*z* 577 (dimer), *m*/*z* 575 (dimer), and *m*/*z* 289 derived from the terminal units. The MS peak at *m*/*z* 407 is also present in the MS spectra and corresponds to the dehydrated structure of the fragment ion formed through the RDA reaction. Taking into account the above and the literature, **peak 9** was identified as a B-type procyanidin trimer [15,34,45].

#### 3.1.4. Flavonols

Two compounds belonging to the flavonol class were identified as quercetin glycosides, **peaks 20** and **21**. **Peak 20** presented an [M−H]^−^ at *m*/*z* 463, which yielded a base peak at *m*/*z* 301, corresponding to the loss of a hexose moiety, together with the characteristic quercetin aglycon ions at *m*/*z* 179 and 151. **Peak 21** presented an [M−H]^−^ ion at *m*/*z* 609 and the characteristic ion corresponding to the quercetin aglycone at *m*/*z* 301, derived from the loss of a molecule of rhamnose and the successive loss of a molecule of glucose [M−H−146−162]^−^. In this sense, compounds **20** and **21** were identified as quercetin-3-*O*-glucoside and quercetin-3-*O*-rutinoside, respectively [29,46].

#### 3.1.5. Anthocyanins

**Peaks 27** and **28** were identified as two cyanidin derivatives. **Peak 27** presented an [M+H]^+^ at *m*/*z* 449 and a fragment ion at *m*/*z* 287 (corresponding to a cyanidin moiety), derived from the loss of a hexose moiety [M−162+H]^+^. Considering the aforementioned observations and the literature, the compound corresponding to **peak 27** was assigned as cyanidin-3-*O*-glucoside [36,47]. **Peak 28** was assigned as a *p*-coumarylated cyanidin derivative, based on the parent ion [M+H]^+^ at *m*/*z* 595 and, equally to **peak 27**, a fragment ion at *m*/*z* 287. **Peak 28** was identified as cyanidin-3-(6′-*p*-coumaroyl) glucoside [48,49,50].

### 3.2. Polyphenol Quantification

**Figure 1:**
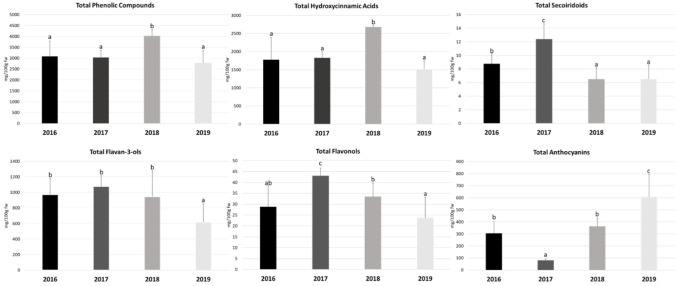
Data obtained as the sum of the concentrations of individual compounds within each class are shown as means ± SD (*n* = 9). Bars with different letters within each bar plot are significantly different at *p* < 0.05 according to the one-way analysis of variance (ANOVA) and Tukey’s multiple range test.

The quantification of the total phenolic compounds in *Prunus lusitanica* fruits as well as the different classes that constitute them, namely, total hydroxycinnamic acids, secoiridoids, flavan-3-ols, flavonols, and anthocyanins, is presented in Figure 1. The quantification of the individual compounds belonging to the different classes is presented in Table 2. The results are expressed as mg/100 g of fruit fresh weight (mg/100g fw) (fruit water content is referred to in Table S1 to perform the unit transformation that allows the comparison of the results with those previously reported in the literature).

Considering the average value of the three different locations in each experimental year (2016–2019) the total content of (poly)phenols ranged from 2772.13 mg/100 g fw in 2019 to 4026.86 mg/100 g fw in 2018. Comparing the four experimental years, the content of total phenolics was only significantly higher in 2018, with no significant differences observed in the remaining three years (Figure 1).

In studies carried out by Mikulic-Petkovsek et al. [19] on fruits belonging to wild *Prunus* species, namely, *Prunus avium*, *Prunus mahaleb*, *Prunus padus*, and *Prunus spinosa*, the values obtained regarding the content of total phenolics were 237.32 mg/100 g fw, 525.17 mg/100 g fw, 1105.33 mg/100 g fw, and 423.48 mg/100 g fw, respectively. The values retrieved in our study, even in the year in which the phenolic content was lower (2019), are considerably higher than the contents referred to in the literature. Moreover, Popović et al. [51] analyzed 15 species of fruits belonging to the *Prunus* genus, reporting values lower than those obtained in this work (although the values were expressed on a dry weight basis), with the highest at 974.79 mg/100 g dw (dry weight) in mahaleb cherry and the lowest at 8.76 mg/100 g dw in purple-leaf cherry plum. In a 3-year study performed by Ruiz-Rodríguez et al. [52] in *Prunus spinosa* fruits, the values concerning the total phenolic content were in good agreement with concentrations obtained in the present work, within the range 1678.99–3797.57 mg/100 g fw in two consecutive seasons. Moreover, in a 2-year experiment in fruits of several sweet cherry (*Prunus avium*) varieties, both local and commercial, carried out by Średnicka-Tober et al. [53], the obtained values ranged from 34.39 to 186.24 mg/100 g fw. Brozdowski et al. [54] obtained *Prunus serotina* fruit (black cherry) values of total phenolic compounds of 1139.40 mg/100 g fw. In a study by Sokół-Łe̜towska et al. [55], the authors reported values of total phenolics ranging from 68.98 to 96.56 mg/100 g fw in *Prunus cerasus* (sour cherry) fruits. In this concern, the observed variations in the diverse classes of phenolic compounds identified in the present work from different seasons and agroclimatic conditions (Table 1) suggest the partial dependence of these compounds on the metabolic stress of plants and thereby on the concentration of the secondary metabolites of the stress response in higher plants [56].

Taking into account the different classes that constitute the phenolic compounds (average values of the four years of study), it was observed that the hydroxycinnamic acids were the class contributing to the highest extent to the (poly)phenolic burden of *Prunus lusitanica* fruits by providing 60.3% of the total phenolics, followed by the flavan-3-ols (27.9%), anthocyanins (10.5%), flavonols (1.0%), and secoiridoids (0.3%). In this regard, similarly, Martini et al. [18] also found that in *Prunus avium* hydroxycinnamic acids represent the majority of the phenolic compounds, followed by flavan-3-ols.

#### 3.2.1. Hydroxycinnamic Acids

Fruits and vegetables are phenolic-acid-rich matrices. They could be coupled via ester, ether, or acetal linkages to plant structural components (lignin, proteins, and cellulose), bigger polyphenols (flavonoids), smaller organic molecules (such as tartaric, quinic, or malic acids and glucose), or other natural products (such as terpenes) [57]. Phenolic acids are mainly divided into two subgroups: derivatives of hydroxybenzoic acid and hydroxycinnamic acid, with the latter being more prevalent than the former. Several studies demonstrate the therapeutic capacity of this set of compounds in the treatment of different pathologies, namely, their anticancer, antidiabetic, neuroprotective, anti-inflammatory, and radioprotective potential [58].

Some fruits in the *Prunus* genus, such as cherries, peaches, plums, and blackthorn, among others, are rich sources of hydroxycinnamic acids, which are the preponderant class of phenolic acids relative to hydroxybenzoic acids [15,34,51,59,60,61,62]. The results obtained in this work confirm this trend for *Prunus lusitanica* fruits, with the hydroxycinnamic acids being the only representative of the phenolic acid group.

Regarding the hydroxycinnamic acid class quantification, the values obtained in this study ranged between 1516.67 and 2678.95 mg/100 g fw (in 2019 and 2018, respectively) (Figure 1). As referred to for total phenolics, the year 2018 stood out from the remaining years of study, with a significantly higher concentration.

In studies carried out on other fruits belonging to the *Prunus* genus, the obtained levels of phenolic acids were much lower than those obtained in this study, which leads us to consider that *Prunus lusitanica* fruits are a valuable source of hydroxycinnamic acids. Głowacka et al. [63] carried out a 4-year study on sour cherry (*Prunus cerasus*) and obtained maximum concentrations of 7.6 mg/100 g fw of phenolic acids. In *Prunus avium*, Mikulic-Petkovsek et al. [19] found a total hydroxycinnamic acid value of 44.24 mg/100 g fw, and Średnicka-Tober et al. [53] obtained, in the same species, values ranging from 35.60 to 49.69 mg/100 g fw. In studies carried out in *Prunus spinosa* fruits, Ruiz-Rodríguez et al. [52] obtained quantification values ranging between 430.32 and 985.56 mg/100 g fw, and Mikulic-Petkovsek et al. [19] obtained a value of 48.78 mg/100 g fw. These last authors also found the amounts of 66.05 mg/100 g fw and 46.13 mg/100 g fw in *Prunus mahaleb* and *Prunus padus*, respectively [19]. In the case of *Prunus laurocerasus* and *Prunus serotina*, the total obtained hydroxycinnamic acid amounts were 145.69–289.39 mg/100 g fw and 22.20 mg/100 g fw, respectively [54,64]. Mihaylova et al. [65] obtained values of phenolic acids in different *Prunus persica* cultivars ranging from 28.72 mg/100 g fw to 786.68 mg/100 g fw.

Concerning the individual profile of hydroxycinnamic acids, of the 21 compounds identified in this class (Table 2), significant differences between years and locations were observed (Table 3). From all the hydroxycinnamic acids identified, the major compounds correspond to **26**, tetra-*O*-acetyl-3-*O*-*p*-coumaroylsucrose isomer; **19**, tri-*O*-acetyl-3-*O*-*p*-coumaroyl sucrose isomer; **13**, di-*O*-acetyl-3-*O*-*p*-coumaroyl sucrose isomer, and **10**, 4-*p*-coumaroylquinic acid (Table 3). These four compounds are responsible (on average) for 82.3%, 75.4%, 81.5%, and 82.3% (in 2016, 2017, 2018, and 2019, respectively) of the total content of hydroxycinnamic acids.

Compound **26** was the phenolic with the highest expression in all years and locations (Table 3), accounting, on average, for 49.2%, 38.8%, 49.8%, and 48.0% (in 2016, 2017, 2018, and 2019, respectively) of the total hydroxycinnamic acid content. Through the analysis of Table 2, it can be verified that the concentration range obtained for this compound was 421.65 to 1462.26 mg/100 g fw, in location 3 in 2016 and 2018, respectively. Although the highest value was obtained for location 3 in 2018, there were no significant differences between this value and the values obtained for the other locations this year. Therefore, it was observed that, for each location, the value obtained in 2018 was significantly higher than those obtained in the other years under study.

For compound **19**, the highest concentration was 487.67 mg/100 g fw in location 1 in 2018, and the lowest was 136.69 mg/100 g fw in location 3 in 2016. Although the highest value was observed in location 1 in 2018, this was not significantly different from the value obtained for location 2 in 2018. However, both were significantly higher than the value obtained in location 3 in 2018. As for compound **26**, in this case, we also verified that, for each location, the value obtained in 2018 was significantly higher than the values obtained in the other years under study. Compound **19** accounted, on average, for 16.5%, 15.6%, 16.3%, and 16.7% (in 2016, 2017, 2018, and 2019, respectively) of the total hydroxycinnamic acid content.

In the case of compound **13**, the values ranged between 109.72 mg/100 g fw in location 3 in 2019 and 300.28 mg/100 g fw in location 3 in 2017. Comparably to location 3, in location 1, it was found that the year 2017 also stood out with the highest value. In location 2, unlike the other two locations assessed in this study, the highest value was observed in 2018, which did not, however, present significant differences compared to the value obtained in 2017. In the case of location 3, the highest value (the year 2017) was significantly different from the remaining years of the study. In location 1, the highest value (the year 2017) was significantly different from the values obtained in 2018 and 2019 but was not significantly different from the value obtained in 2016. For location 2, the highest value (the year 2018) was not significantly different from the value obtained in 2017, although both were significantly higher than those obtained in 2016 and 2019. This compound accounted, on average, for 11.2%, 15.2%, 9.1%, and 10.0% (in 2016, 2017, 2018, and 2019, respectively) of the total hydroxycinnamic acid content.

Isomers of the compounds assigned in this study as acetyl-*p*-coumaroylsucroses (**peaks 3**, **11**–**19**, and **22**–**26**) (Table 2) have already been identified in *Prunus mume* flowers and fruits [29,41,42,66,67,68,69,70,71,72], although, to date, none of those addresses their quantification, preventing us from making a comparison with the amounts obtained for *Prunus lusitanica*.

The concentrations recorded for compound **10** (4-*p*-coumaroylquinic acid) ranged from 72.31 to 177.24 mg/100 g fw in location 3 in 2016 and 2018, respectively (Table 2). Similar to compound **26**, for compound **10** the year 2018 stood out in all locations, with significantly higher values compared to the other years in the same location. In turn, there were no significant differences between the values obtained in 2018 in the different locations.

Comparing the concentrations recorded for compound **10** (4-*p*-coumarouylquinic acid) in this study with the levels reported by other authors concerning different fruits of other *Prunus* species, it was stressed that the amounts obtained in *Prunus avium* were quite variable, with values of 53 mg/100 g fw in studies performed by Bastos et al. [73]. On the other hand, Martini et al. [18] obtained amounts of 4-*p*-coumarouylquinic acid ranging between 0.74 and 18.58 mg/100g fw, and Mikulic-Petkovsek et al. [19] obtained average values of 1.14 mg/100 g fw. Brozdowski et al. [54], in *Prunus serotina* fruits, obtained values of 1.64 mg/100 g fw.

Contrary to observations of several species belonging to the *Prunus* genus in which neochlorogenic acid (3-*O*-Caffeoylquinic acid) has been labelled as the most abundant phenolic acid [15,32,51,57,61,74,75], in our study, this representative showed very low expression when compared to the aforementioned compounds, being one of those with the lowest concentration, presenting expression of 0.1 in 2016, 2017, and 2018 and 0.2% in 2019.

#### 3.2.2. Secoiridoids

Iridoids are a large class of cyclopentane [c] pyran monoterpenoids found in plants and insects that have positive health effects. In this frame, secoiridoids are produced when the cyclopentane ring of iridoids is broken, triggering the biological effects associated with anti-inflammatory, immunosuppressive, antidiabetic, neuroprotective, anticancer, and anti-obesity activities [76].

This class of compounds as well as their biological properties have already been identified and studied in other berry-like fruits, such as *Cornus officinalis* [77,78], *Lonicera* spp. [79], *Ligustrum japonicum* [80], and *Ligustrum lucidum* [81]. In the present work, compound **6**, identified as 6′-*O*-*β*-D-glucosyl swertiamarin, was the only secoiridoid identified, and as far as we know, has never been identified in the *Prunus* genus or other fruits.

Regarding its quantification and considering the average value of the three locations in each year under study, as shown in Figure 1, this compound had a significantly higher expression in the year 2017 compared to the other three years, with a maximum value of 12.40 mg/100 g fw. The minimum value of 6.51 mg/100 g fw was recorded in the year 2018. Moreover, Figure 1 shows that the total contents of secoiridoids obtained in 2018 and 2019 were not significantly different.

Regarding the data from Table 3, analyzing the values obtained in each location and year separately, it is possible to verify that the values ranged between 4.23 mg/100 g fw for location 3 in 2019 and 14.98 mg/100 g fw for location 3 in 2017.

The highest concentrations of this compound, regardless of location, were reached in 2017, always with significantly higher values than those obtained in the remaining years of the study for each location. Moreover, it can be observed that in 2017 all values were significantly different between the three locations.

Considering the changes in the concentration of this compound in the different locations evaluated, in location 1 there were no significant differences between the years 2016 and 2017 or between 2018 and 2019. On the contrary, in location 2 the values regarding the four experimental years were all significantly different. Regarding location 3, it was verified that the values obtained for 2016 and 2018 were not significantly different from each other but were significantly different from 2017 and 2019.

#### 3.2.3. Flavan-3-ols

Compounds belonging to the class of flavan-3-ols can be divided into simple flavan-3-ols, alkaloid flavan-3-ols, and oligomeric flavan-3-ols [82]. This is a class of compounds that has been extensively studied and whose biological properties such as antioxidant, neuroprotective, anticancer, anti-inflammatory, cardioprotective, antimicrobial, and antiallergic, and antidiabetic activities, among others, are widely recognized [83,84].

Flavan-3-ols are the second most abundant group of phenolic compounds in *Prunus lusitanica* fruits. Their levels ranged from 617.49 (the year 2019) to 1075.95 mg/100g fw (the year 2017), even though there were significantly lower values compared with the other years (that were not significantly different from each other) only in 2019 (Figure 1). These results indicate that *Prunus lusitanica* fruits can be considered rich sources of these compounds in comparison with other *Prunus* species, for example, *Prunus serotina* (black cherry), with values of 701.70 mg/100 g fw [54]; *Prunus padus* (bird cherry) at 12.41 mg/100 g fw; *Prunus avium* (sweet cherry) at 54.60 mg/100 g fw [19], and *Prunus cerasus* (sour cherry) with values ranging from 2.87 to 63.08 mg/100 g fw [55].

Two representatives of this class were identified in this study (Table 2), catechin (**peak 8**) and a B-type procyanidin trimer (**peak 9**). For compound 8, the quantification values ranged from 11.53 mg/100 g fw in location 2 in 2018 to 30.57 mg/100 g fw in location 3 in 2017 (Table 3).

When analyzing the different locations, it was observed that, in the cases of the location 2 and location 3, the highest values were obtained in 2017. For location 1, the highest value was observed in 2019, although these results were not significantly different from the value obtained in 2017. We can also verify that in the location 1 there were significantly lower values than those obtained in the other years only in 2016. In location 3, only in 2017, the values were significantly higher. It is also worth mentioning that for this location, in the year 2018, this compound was not detected.

As for location 2, we can observe that the values for the year 2017 were not significantly different from those of 2019, which, in turn, were not significantly different from 2016, and that the latter were not significantly different from 2018.

This compound accounted for 1.5%, 2.2%, 1.1%, and 2.9% of the total amount of flavan-3-ols in 2016, 2017, 2018, and 2019, respectively.

Bayrambaş et al. [85] found, in different varieties of *Prunus laurocerasus* fruits, values ranging between 0.03 and 23.30 mg/100g fw. A similar trend was obtained by Nunes et al. [86] when analyzing plum fruits (*Prunus domestica*), with minimum values between 1.50 and 22.00 mg/100 g fw. Brozdowski et al. [54], in *Prunus serotina*, obtained catechin values of 30.30 mg/100 g fw. In *Prunus humilis* fruits, Fu et al. [87] obtained catechin values ranging between 15.76 and 120.81 mg/100 g fw. Guo et al. [88] characterized the flavan-3-ols contents of peaches (*Prunus persica*) and nectarines (*Prunus persica* Var. Nectarica), reporting catechin values in the range of those obtained by us, namely, between 2.76 and 116.97 mg/100 g fw. Donno et al. [89] found, in *Prunus padus* fruits, catechin amounts of 56.66 mg/100 g fw. Moreover, in *Prunus spinosa*, *Prunus armenica*, and *Prunus padus*, some authors obtained lower concentrations of catechin than those obtained in our research, namely, 0.64, 0.30–7.50, and 2.89–3.41 mg/100 g fw, respectively [8,90,91].

In the case of compound **9**, the values ranged from 338.88 mg/100g in location 3 in 2019 to 1302.16 mg/100 g in location 2 in 2018 (Table 3). In the case of this compound, unlike the previous one, the highest values were obtained in different years for each location.

For location 1, the highest value was obtained in 2016, which was significantly different from the values obtained in the remaining years. These, in turn, were not significantly different from each other.

Regarding location 2, the highest value was obtained in 2018, which was significantly different from all the others. However, the values obtained in 2016 and 2017 were not significantly different from each other but were significantly different from the value obtained in 2019.

In location 3, the highest value was obtained in 2017, which was significantly different from the other years. The values obtained in 2016 and 2018 were not significantly different from each other but were significantly different from the value obtained in 2019. It is also important to note that the lowest values were always observed in 2019 in all locations.

**Peak 9** accounted for values of 98.5%, 97.8%, 98.9%, and 97.1% in 2016, 2017, 2018, and 2019, respectively. It is also worth mentioning that the B-type procyanidin trimer was, along with compound **26**, the compound with the highest expression of all the phenolic compounds found.

Most of the berry-like fruits are well-known to contain high amounts of procyanidins [92]. The contents of procyanidins in our study were quite remarkable. Tomić et al. [93] observed that among the tested groups of phenolic compounds, the level of flavan-3-ols were the highest and that the major compound from this class was a procyanidin trimer, resembling the results obtained in our study, although the values were much lower, with a maximum of 25.30 mg/100 g fw. Brozdowski et al. [54] obtained in *Prunus serotina* amounts of procyanidin trimers (sum of four) of 192.90 mg/100g fw. Mikulic-Petkovsek et al. [19] found the presence of procyanidin oligomers up to tetramers in *Prunus* species under study, and similar to our case, the only representative of this class in *Prunus spinosa* was a procyanidin trimer with values of 1.47 mg/100 g fw. Higher values were found by these authors in *Prunus avium*, with procyanidin trimers contents (sum of three) reaching 15.52 mg/100 g fw. Moreover, Wojdyło et al. [50] verified that *Prunus cerasus* was rich in procyanidins, especially procyanidin trimers, and obtained procyanidin contents ranging from 403.57 mg/100 g dw to 1215.67 mg/100 g dw. However, it has to be taken into consideration that these authors expressed their results on a dry weight basis, which implies considerably lower values than if they had been expressed in fresh weight. In a study carried out by Ayour et al. [91] in different cultivars of apricot (*Prunus armenica*), the obtained values of procyanidins varied between 3 mg/100 g fw and 141.10 mg/100 g fw. Lower values were obtained by Iglesias-Carres et al. [94] concerning both a procyanidin trimer (0.04 mg/100 g dw) and the total procyanidin content (0.32 mg/100 g dw). In *Prunus persica* var. nucipersica fruits, Tomás-Barberán et al. [95] found a maximum total procyanidin content of 63.60 mg/100 g fw. Liaudanskas et al. [96] studied the phenolic contents of several plum cultivars (*Prunus domestica* and *Prunus cerasifera*) and obtained a maximum procyanidin value (sum of procyanidin A2 and procyanidin C1) of 165.86 mg/100 g dw.

Wojdyło et al. [47] found very high values of polymeric procyanidins in peach kernels, with contents ranging from 2680 mg/100 g dw to 10,980 mg/100 g dw.

Procyanidin monomers, dimers, and trimers are absorbed into the blood system to a much larger extent than larger oligomers and polymers, and a polymerization degree below 4 implies relatively large amounts of short-chain procyanidin species that are more readily absorbed, making *Prunus lusitanica* fruits, resembling *Prunus cerasus* fruits, a particularly good source of short-chain procyanidins [97].

#### 3.2.4. Flavonols

In higher plants, flavonols are equally distributed throughout the fruits, flowers, leaves, and stems. Quercetin is the flavonol that has been studied the most [98]. According to scientific research, compounds belonging to the flavonol group, particularly quercetin and its glycosides, have a wide range of biological effects, such as reducing the risk of cardiovascular illness, metabolic disorders, and certain types of cancer [99,100,101,102,103].

The flavonols correspond to the second class of phenolics with lower representation in *Prunus lusitanica* fruits. Regarding their quantification, through the analysis of Figure 1, it can be observed that the average contents obtained in our study ranged from 23.80 mg/100 g fw in 2019 to 43.08 mg/100 g fw in 2017. In this case, the levels obtained in 2017 were significantly higher than those obtained in the remaining years of the study. In 2019, although the values were lower than the others, the observed differences were not statistically significant.

In the research of Ruiz-Rodríguez et al. [52] with *Prunus spinosa* fruits, the values of total flavonols were higher than those obtained in our study, with minimum amounts of 87.63 mg/100 g fw and a maximum of 226.69 mg/100 g fw. In *Prunus mahaleb* fruits, the values referring to total flavonols ranged between 20.00 and 30.00 mg/100 g fw [104]. In the quantification of total flavonols in *Prunus serotina* (black cherry) fruits, the levels obtained by Brozdowski et al. [54] were 12.30 mg/100 g fw and were therefore lower than those obtained in *Prunus lusitanica* fruits.

Regarding the profiles of individual compounds within this class, two quercetin derivatives were identified (Table 2), namely, quercetin-3-*O*-glucoside (**peak 20**) and quercetin-3-*O*-rutinoside (**peak 21**). Other authors, in different species of fruits belonging to the genus *Prunus*, such as *Prunus domestica*, *Prunus avium*, *Prunus cerasus*, *Prunus serotina*, and *Prunus armenica*, and similar to what was observed in this study, verified that the quercetin derivatives were the major representatives of the flavonol class, with quercetin-3-*O*-rutinoside being the one with the highest expression [18,54,74,93,105,106,107].

Analyzing the quantification data for compound **20** (Table 3), it was found that the obtained values varied between 0.75 mg/100 g fw for location 3 in 2019 and 6.31 mg/100 g fw for location 2 in 2018. All the same, the highest values were obtained in 2017 for location 1 and location 3. In the location 2, as previously mentioned, the highest value was in 2018; however, this was not significantly higher than the value obtained in 2017.

Analyzing the locations individually, the highest value for location 1 in 2017 was found to be significantly higher than the others, and these in turn were not significantly different from each other. In location 2, the value obtained in 2018 was not significantly different from that of 2017, both of which were significantly different from the values obtained in 2016 and 2019, and the latter two were not significantly different from each other. In location 3, all values were significantly different from each other.

Compound **20** had a percentage of expression, compared to the total flavonol contents, of 8.1%, 10.4%, 8.7%, and 8.0% in the years 2016, 2017, 2018, and 2019, respectively.

In the case of compound **21**, the obtained values ranged between 13.40 mg/100 g fw in location 3 in 2019 and 41.70 mg/100 g fw in location 1 in 2017. The highest levels were recorded in 2017 in the three locations, and these showed no significant differences between them.

Considering each location independently, it was possible to verify that in location 1 the value for 2017 was significantly higher than the remaining values and the levels obtained in 2016 and 2019 were not significantly different from each other, but both were significantly higher than that obtained in 2018 (Table 3). In location 2, all values were significantly different from each other. For location 3, the levels for 2017 and 2016 were not significantly different from each other, but they were significantly different from the values obtained in 2018 and 2019, which in turn were also significantly different from each other.

The contribution of compound **21** to the total content of flavonols was 91.9%, 89.6%, 88.7%, and 92.0% in 2016, 2017, 2018, and 2019, respectively. Because of these results and considering those mentioned above for compound **20**, we can verify that the contribution of compound **21** was much higher.

Comparing the values reported in the literature with those obtained in this study, it was observed that in studies carried out with *Prunus avium* fruits, the levels referring to quercetin-3-*O*-glucoside were within the range of values obtained in our study, namely, 0.39–26.55 mg/100 g fw [18]. Średnicka-Tober et al. [53] tended to have lower levels (0.21–1.36 mg/100g fw), and the same was observed for the content of quercetin-3-*O*-rutinoside (0.24–2.77 mg/100 g fw) obtained by these authors. Martini et al. [18] and Gao et al. [74] reported contents of quercetin-3-*O*-rutinoside of 5.19–51.97 and 0.52–39.82 mg/100 g fw, respectively, in good agreement with the concentrations reported in the present work. In *Prunus spinosa*, a quercetin-3-*O*-glucoside content of 3.20 mg/100 g fw was obtained by Radovanović et al. [90], which is in agreement with that obtained in our study. In the case of quercetin-3-*O*-rutinoside, the values obtained in this species by several authors were significantly lower than those obtained by us, namely, 1.39 mg/100 g fw [90], 4.86 mg/100 g fw [8], and 4.67 mg/100 g fw [108]. Blando et al. [104] obtained levels of quercetin-3-*O*-glucoside ranging from 9.00–17.00 mg/100 g fw and of quercetin-3-*O*-rutinoside from 11.00–17.00 mg/100 g fw in *Prunus mahaleb*. The first result was higher than those obtained by us in *Prunus lusitanica* fruits, and the second was lower. Compared to *Prunus domestica* fruits, the levels of quercetin-3-*O*-rutinoside (11.00–43.00 mg/100 g fw) and quercetin-3-*O*-glucoside (2.70–13.3.0 mg/100 g fw) were similar to those obtained in this study [86]. Other authors obtained considerably lower levels of quercetin-3-*O*-rutinoside (1.92 mg/100 g fw) in the same species [108]. Brozdowski et al. [54] obtained lower values than ours in *Prunus serotina* fruits, either for quercetin-3-*O*-glucoside (1.00 mg/100 g fw) or for quercetin-3-*O*-rutinoside (13.55 mg/100 g fw). Different quercetin-3-*O*-rutinoside levels in *Prunus padus* fruits were reported by different authors. Cosmulescu et al. [8] obtained levels (6.67mg/100 g fw) considerably lower than ours, while Donno et al. [89] obtained values (26.67 mg/100 g fw) that were in line with those obtained in our study. The same trend was observed by Guo et al. [88] regarding quercetin-3-*O*-rutinoside in *Prunus persica* fruits, with levels ranging from 0.62 to 6.62mg/100 g fw.

Considering the data obtained by the different authors in the different species mentioned above and our results, we can say that *Prunus lusitanica* fruits are good sources of flavonols, particularly quercetin-3-*O*-rutinoside.

#### 3.2.5. Anthocyanins

Anthocyanins are a ubiquitous class of flavonoids that are synthesized from the flavonoid pathway through the condensation of anthocyanidins and sugars [109]. Those compounds present a wide range of biological effects, including antioxidant and anti-inflammatory properties as well as the ability to protect against age-related chronic diseases such as cancer, cardiovascular diseases, and ocular and neurological disorders. They also showed antiviral properties [110,111].

Regarding the average content of anthocyanins obtained in each year of the study, it can be seen, through the analysis of Figure 1, that the minimum obtained content was 81.44 mg/100 g fw in 2017 and the maximum content was 607.62 mg/100 g fw in 2019. In addition, it was also verified that the obtained levels were significantly different in all years.

The levels of anthocyanins found in the literature are quite variable among fruits belonging to the *Prunus* genus. In *Prunus humilis*, Liu et al. [112] obtained values ranging between 69 and 152 mg/100 g fw, while Fu et al. [87] obtained levels between 15.24 and 231.18 mg/100 g fw for the same species. In *Prunus padus*, a concentration of 207.12 mg/100 g fw was observed by Mikulic-Petkovsek et al. [19], which is in the same range as those previously reported in *Prunus humilis*. In studies carried out on *Prunus spinosa*, Mikulic-Petkovsek et al. [19] recorded total amounts of 233.46 mg/100 g fw, whereas Ruiz-Rodríguez et al. [52] obtained a maximum content about 10 times higher (2585.00 mg/100 g fw) in the same species. Moreover, high values were recorded in *Prunus serotina* (402.00 mg/100 g fw) by Brozdowski et al. [54] and in *Prunus mahaleb* (between 260.00 and 550.00 mg/100 g fw) by Blando et al. [104]. Taking into account the great variability observed by us between the years under study, the levels of anthocyanins obtained in *Prunus lusitanica* fruits were in line with the range of values obtained in the species discussed above. Distinct from the previously mentioned species (including *Prunus lusitanica*), are *Prunus avium* fruits, for which different authors reported considerably lower levels, 3.99–17.75 mg/100 g fw and 57.10 mg/100 g fw [19,113]. Moreover, the total levels of anthocyanins obtained in our study are in the range of those obtained in other berry-like fruits belonging to different genera, such as Vaccinium, Ribes, and Rubus, known for their high levels of anthocyanins and the beneficial effects that are associated with them [114,115,116,117].

Two cyanidin derivatives belonging to the anthocyanins class were identified in this study (Table 2): compound **27,** identified as cyanidin-3-*O*-glucoside, and compound **28,** identified as cyanidin-3-(6-*trans*-*p*-coumaroyl)glucoside.

Regarding the quantification of compound **27** (Table 3), the values ranged from 41.03 mg/100 g fw in location 1 in 2017 to 450.54 mg/100 g fw in location 1 in 2019. However, although the highest levels were always recorded in 2019 for all locations, only the levels obtained in location 1 in 2019 were significantly higher than those obtained in the other locations for the same year.

Taking into account the individual locations, it can be seen that in location 1 and location 2 the levels obtained in the different years were always significantly different. Nonetheless, in the case of location 3, it was found that the levels obtained in the years 2016 and 2018 were not significantly different from each other, although they were significantly different from the values of 2017 and 2019.

From all anthocyanins, compound **27** contributed 54.4%, 58.4%, 33.9%, and 55.5% in 2016, 2017, 2018, and 2019, respectively. For this anthocyanin (cyanidin-3-*O*-glucoside), in comparison with the levels obtained in other fruits of the genus *Prunus*, a division into two groups can be performed: a first, with levels lower than those obtained by us in *Prunus lusitanica*, represented by *Prunus avium*, with values ranging between 1.42 and 6.36 mg/100 g fw [19,53,112], and a second, composed of those with levels within the range of those obtained in this study, namely, *Prunus padus* with 150.15 mg/100 g fw, *Prunus mahaleb* with 89.86 mg/100 g fw, *Prunus spinosa* with 128.65 mg/100 g fw [19], and *Prunus serotina* with 213.80 mg/100 g fw [54]. Despite this classification, it is important to consider that other anthocyanins besides cyanidin-3-*O*-glucoside, such as other derivatives of cyanidin, pelargonidin, petunidin, and peonidin, contribute to the quantitative profile of this phenolic class in the species mentioned above.

For compound **28**, quantification values (Table 3) ranged from 31.35 mg/100 g fw in location 1 in 2017 to 369.70 mg/100 g fw in location 1 in 2019. As for compound **27**, the highest levels were also always recorded in 2019 for all locations, and the levels obtained in location 1 in 2019 were significantly higher than those obtained in the other three locations for the same year. The only behavioral difference between compounds **27** and **28** lies in the fact that, for the location 3 and contrary to compound **27**, for the contents of compound **28** there were no significant differences between the years 2016, 2018, and 2019.

In addition to it is also worth mentioning the fact that for both compounds (**27** and **28**) the lowest levels were always reached in 2017 in the three locations under study.

Compound **28** accounted for 45.6%, 41.6%, 45.5%, and 45.0% of the total anthocyanin contents with in 2016, 2017, 2018, and 2019, respectively.

For compound **28** (cyanidin-3-(6-*trans*-*p*-coumaroyl)glucoside), its description in the *Prunus* genus, namely, *Prunus cerasus* [50], has been scarcely reported, with a concentration of 0.10 mg/100 g dw, much lower than the concentration found in *Prunus lusitanica* in the present work (Table 3). In addition to *Prunus cerasus*, this compound was also quantified in grapes, either table grapes [48] or different red grape varieties for wine production [118], with contents of 0.33–1.24 mg/100g fw and 28–85 mg/100 g fw, respectively.

### 3.3. Antioxidant Capacity of Prunus lusitanica Fruits

Free radicals can damage cells and cause disease and ageing. Accordingly, the ability of substances to scavenge radicals is beneficial in terms of human health [119]. Thus, there is currently a growing need to find new sources of antioxidant compounds that could assist in the defense against free radicals [120].

From the different analytical approaches available to assess the antioxidant capacity, three in vitro methods, the ABTS, DPPH, and FRAP, were used to evaluate the antioxidant capacity of *Prunus lusitanica* fruits grown under different conditions (locations and season) (Table 4).

The ABTS assay yielded values ranging from 7.88 mmol TE/100 g fw in location 3 in 2019 to 10.69 mmol TE/100 g fw in location 2 in 2017. For the DPPH assay, the results ranged from 5.18 mmol TE/100 g fw in location 3 in 2019 to 8.17 mmol TE/100 g fw in location 2 in 2017. Concerning the FRAP assay, values ranged from 8.76 mmol TE/100 g fw in location 3 in 2016 to 11.76 mmol TE/100 g fw in location 1 in 2018.

Analyzing Table 4 and considering the antioxidant capacity values obtained through the different methods in the three locations, it was verified that for both DPPH and ABTS, the values obtained in 2017 were always significantly higher than those obtained in the remaining years of the study, except for the one registered in location 1 in 2018 for ABTS, which was not significantly different from that obtained in 2017. In the case of FRAP, there was a different trend, with the values obtained in 2018 standing out, with values significantly higher than those obtained in the remaining years in each location. However, an exception was observed for location 2 in 2017, whose values were not significantly different from those obtained in 2018 in the same location. In addition, we can also verify that the values obtained for FRAP in 2017 were the second highest values in all locations and were significantly different from those obtained in 2016 and 2019.

The highest values, regardless of location, as mentioned above, were always obtained in the same year for the three methods used (ABTS and DPPH in 2017 and FRAP in 2018). Comparing the highest values between locations, it was observed that in 2017 that obtained in location 2 was not significantly different from that obtained in location 1, but it was different from that obtained in location 3. However, location 1 and location 3 did not show significant differences between them. In the case of DPPH, the values obtained in location 2 and location 3 were not significantly different from each other, but both were significantly higher than those obtained in location 1. Regarding FRAP, the value obtained in location 1 in 2018 was not significantly different from the value obtained in location 3. However, both were significantly different from the value obtained in location 2 (Table 4).

Several studies have reported the antioxidant capacity of different fruits from *Prunus* species. In the year 2012, Wang and collaborators reported for *Prunus cerasifera* the range of values between 1.12 and 4.50 mmol TE/100g fw for the FRAP assay [121]. Moreover, Blando et al. [104] found for *Prunus mahaleb* fruits the value of 4.50 mmol TE/100g fw using the ABTS method, and in a study performed by Martini et al. [18] with *Prunus avium*, they related values between 0.53 mmol TE/100 g fw and 0.32 mmol TE/100 g fw for the FRAP assay and between 1.32 mmol TE/100 g fw and 6.79 mmol TE/100 g fW for the ABTS assay. Luna-Vázquez et al. [122] obtained, for *Prunus serotina* fruits, the values of 1.46 mmol TE/100 g fw for the FRAP assay and 2.06 mmol TE/100 g fw for the DPPH assay. Some authors performed studies with *Prunus spinosa* fruits and found values for the ABTS assay ranging from 1.83 mmol TE/100 g fw to 7.64 mmol TE/100 g fw, for the FRAP assay ranging from 7.11 mmol TE/100 g fw to 15.17 mmol TE/100 g fw [52], and for the DPPH assay of 0.26 mmol TE/100 g fw [8]. A study performed by Ozturk et al. [123] with *Prunus laurocerasus* fruits presented for the ABTS, DPPH, and FRAP assays the range values of 1.76–2.32 mmol TE/100 g fw; 3.09–4.35 mmol TE/100 g fw, and 1.22–1.47 mmol TE/100 g fw, respectively. Altuntas et al. [124] obtained, for the same species, the values of 0.34 mmol TE/100 g fw for FRAP and 0.37 mmol TE/100 g fw for ABTS. For some studies with *Prunus padus*, the authors reported, for the DPPH assay, the value of 2.95 mmol TE/100 g fw [8] and the value of 3.15 mmol TE/100g fw for the FRAP assay [122].

When evaluating the in vitro antioxidant capacity of *Prunus persica*, some authors obtained DPPH values between 0.03 mmol TE/100 g fw and 0.73 mmol TE/100 g fw and the value of 0.10 mmol TE/100 g fw. For ABTS, they obtained values between 0.13 mmol TE/100 g fw and 1.33 mmol TE/100 g fw and the value of 0.07 mmol TE/100 g fw. Regarding the FRAP assay, the authors reported values between 0.09 mmol TE/100 g fw and 1.10 mmol TE/100 g fw and the value of 0.34 mmol TE/100 g fw [65,88].

Analyzing the antioxidant capacity results obtained in *Prunus lusitanica* fruits, it was found that they were comparable, tending to be higher than those reported in other species of the same genus.

### 3.4. Correlation between the Phenolic Compounds and the Antioxidant Capacity

To explore the relationship between the antioxidant capacity and phenolic content in *Prunus lusitanica* fruits, a correlation analysis was carried out. The results of a Pearson correlation analysis (Table S2) showed that the individual phenolic contents in *Prunus lusitanica* fruits correlated positively with the antioxidant capacity accessed through different methodologies, which implied that the antioxidant capacity of those fruits is mainly attributed to its phenolic composition. In this regard, a highly positive correlation between DPPH and ABTS was obtained in our study (*r* = 0.861, *p* < 0.001). Similar results were obtained by other authors in other fruits belonging to the *Prunus* genus, namely, peaches, nectarines, plums, and apricots [125,126,127]. Regarding the correlation of FRAP with ABTS and DPPH (*r* = 0.529 and *r* = 0.523, respectively), it was verified that they were not highly positively correlated as in the case of ABTS with DPPH, in agreement with the previously reported observations by other authors [128,129]. These results can be attributed to the different characteristics and mechanisms of action inherent to the different methods used to determine the antioxidant capacity [130].

From the 21 compounds belonging to the hydroxycinnamic acid class, it was found through the retrieved correlation values (Table S2) that 16 of them are positively correlated (with a probability of at least 95%) with at least one of the three methods used in the evaluation of the antioxidant capacity of *Prunus lusitanica* fruits.

Regarding the correlation of DPPH with hydroxycinnamic acids, this was highlighted by the existence of highly significant correlations (*p* < 0.001) with four of these compounds, namely, **11**, **13**, **15**, and **24** (*r* = 0.866, 0.870, 0.899, and 0.852, respectively), all of them assigned as acetyl-*p*-coumaroylsucrose derivatives; strongly significant correlations (*p* < 0.01) with three of them, namely, 3, 5, and 7 (*r* = 0.776, 0.845, and 0.837, respectively); and significant correlations (*p* < 0.05) with compounds 2, 12, 14, and 22 (*r* = 0.689, 0.691, 0.708, and 0.645, respectively). The ABTS-based antioxidant capacity presented significant positive correlations (*p* < 0.05) with six hydroxycinnamic acids, namely, **5**, **7**, **11**, **13**, **14**, and **24** (*r* = 0.625, 0.710, 0.693, 0.692, 0.707, and 0.664, respectively), and a strong positive correlation (*p* < 0.01) with compound **15** (*r* = 0.797). Regarding FRAP, it showed a strong positive correlation (*p* < 0.01) with six hydroxycinnamic acids, namely, compounds **3**, **10**, **22**, **23**, **25**, and **26** (*r* = 0.724, 0.790, 0.743, 0.739, 0.734, and 0.725, respectively), and a significant positive correlation with three of them, namely, compounds **15**, **19**, and **24** (*r* = 0.619, 0.671, and 0.645, respectively).

The evident strong correlations between hydroxycinnamic acids and the antioxidant capacity of *Prunus lusitanica* fruits can be attributed to the fact that the phenolic acids are recognized as chain-breaking antioxidants that act through radical scavenging activity, which is correlated with their capacity to donate hydrogen or electrons and their capacity to delocalize/stabilize the resulting phenoxyl radicals within their structure [131]. In the particular case of hydroxycinnamic acids, including those identified in this study, their particular antioxidant capacity can be explained at a structural level by the stabilization of the aromatic ring, attributed to the presence of –CH=CH–COOH (propenoic side chain) and OH groups in the aromatic ring, which contributes to the electron-donating effect that improves the hydrogen-donating capacity in the reaction with free radicals [132].

Ding et al. [133] found that phenolic acids present in *Prunus persica* fruits were significantly related to their radical scavenging capacity. Similar results were observed by several authors in *Prunus avium* and *Prunus cerasus* fruits, which verified that the presence of hydroxycinnamic acids markedly contributed to their antioxidant capacity and, more specifically and similar to what was seen in our study, the correlation between the *p*-coumarouylquinic acid and coumaric acid derivatives with antioxidant capacity [50,134,135,136]. As verified by us for compounds **11** and **12**, in studies carried out by Yan et al. [137] in *Prunus mume* fruits, the mono-*O*-acetyl-3-*O*-*p*-coumaroylsucroses also presented a strong antioxidant capacity.

The only compound belonging to the secoiridoids class (compound **6**) presents significant positive correlations (*p* < 0.05) with the ABTS and DPPH methods (*r* = 0.600 and 0.671, respectively). Unfortunately, we did not find studies in the literature that allow us to compare these results.

Of the two compounds identified in the flavan-3-ols class (**8** and **9**) (Table 2), a strong positive correlation (*p* < 0.01) was found between the B-Type procyanidin trimer (compound **9**) and DPPH (*r* = 0.761). It has been demonstrated that flavan-3-ols and their polymeric condensation products, proanthocyanidins, act as antioxidants through a variety of endogenous and exogenous mechanisms, with the main one being breaking the free radical chain reaction, since the substantial electron delocalization caused by the catechol unit on the aromatic B-ring gives the corresponding oxidized forms greater stability. However, this antioxidant activity also includes other possible pathways when the procyanidins are present in a biological medium, such as the capacity to chelate transition metals, the ability to inhibit the production of any additional pro-oxidants, or the ability to work in concert with other antioxidants. Additionally, some structural features, such as the number of hydroxyl groups, methoxy esters, double bonds, and carbohydrate moieties, modulate the antioxidant capacity of those compounds. However, the galloyl (galloylation) and phenolic units (polymerization) contents are likely the most significant ones, once their impact on the different antioxidant mechanisms is substantial [138,139]. Studies carried out by several authors have shown that an increase in the degree of polymerization implies an increase in the antioxidant capacity (monomer < dimer < trimer < tetramer < pentamer < hexamer) [140,141,142]. This may explain the fact that, in our study, a significant correlation of compound **8** (catechin) with the antioxidant capacity was not observed, contrary to what was observed for compound **9** (procyanidin trimer). Plumb et al. [143] observed that an increase in antioxidant activity was obtained from monomer to trimer and subsequently decreased from trimer to tetramer. Similar results were obtained by Shahat et al. [144], who found that the B-type procyanidin trimer showed higher antioxidant activity compared to larger oligomers. Moreover, in studies carried out by Zhang et al. [145] and Wojdyło et al. [50] on *Prunus padus* and *Prunus cerasus* fruits, respectively, it was found that the antioxidant capacity was directly related to the proanthocyanidins present.

Regarding the compounds belonging to the anthocyanin class (compounds **27** and **28**), negative correlations were obtained (Table S2) for both compounds in the three methods used to assess the antioxidant capacity, particularly DPPH, with significant negative correlations (*p* < 0.05) of *r* = −0.646 and *r* = −0.529 for compounds 27 and 28, respectively.

In a study performed by Ruiz-Rodríguez et al. [52] in *Prunus spinosa* fruits, they also found the same negative correlation between the anthocyanin content and the DPPH assay. The same negative correlation of anthocyanins with the antioxidant capacity was also verified by Gonçalves et al. [135] in a study performed with Portuguese cherries. Guimarães et al. [60] observed, in a study with *Prunus spinosa* fruits, that the samples with the highest contents of anthocyanins showed the lowest antioxidant capacity, which was attributed to a potential pro-oxidant effect of these compounds. The same type of result was also reported by other authors concerning anthocyanins in some berry fruits known for their high anthocyanin contents. Szymanowska and Baraniak [146] reported, in their study with raspberry pomace, the same negative relationship between anthocyanin content and the DPPH assay. Rigolon et al. [147] obtained, in their research with fruits of Rubus sp (blackberry) and Vaccinium sp (blueberry), species known for their high anthocyanin contents, a negative correlation between the anthocyanin contents and the ABTS, DPPH, and FRAP assays, similar to our study.

About compound **20** (Table 2), this compound showed a significant positive correlation (*p* < 0.05) with ABTS (*r* = 0.613) and a strong positive correlation (*p* < 0.01) with DPPH (*r* = 0.729). Compound **21**, on the other hand, showed a significant correlation (*p* < 0.05) only with DPPH (*r* = 0.631).

Flavonols are widely distributed in plants, being typically found in the glycosylated form [98], as was the case of the two identified in this study, which, due to their structural features, confers to these compounds a high antioxidant capacity, namely, the presence of a 4-keto function conjugated with the 2,3 double bond and the hydroxyl groups in the B ring, especially 3-OH, once the oxygen in this group presents the smallest electron density, is easily ionized [42,57].

In line with the results obtained in our study, high positive correlations between quercetin-3-*O*-rutinoside and DPPH were found in studies performed with *Prunus humilis* fruits [148]. Similar results were also obtained with *Prunus armenica* and *Prunus pseudocerasus* fruits [149,150,151]. In addition to these, other authors have verified in their studies on *Prunus avium* that the high antioxidant capacity of these fruits is mainly attributed to the flavonoid content, particularly quercetin-3-*O*-rutinoside [14].

However, in other species, or even analyzing the antioxidant activity of the pure compound, other authors reported significant correlations between quercetin-3-*O*-glucoside and the antioxidant capacity by the ABTS and DPPH methods [152,153,154].

### 3.5. Principal Component Analysis

**Figure 2:**
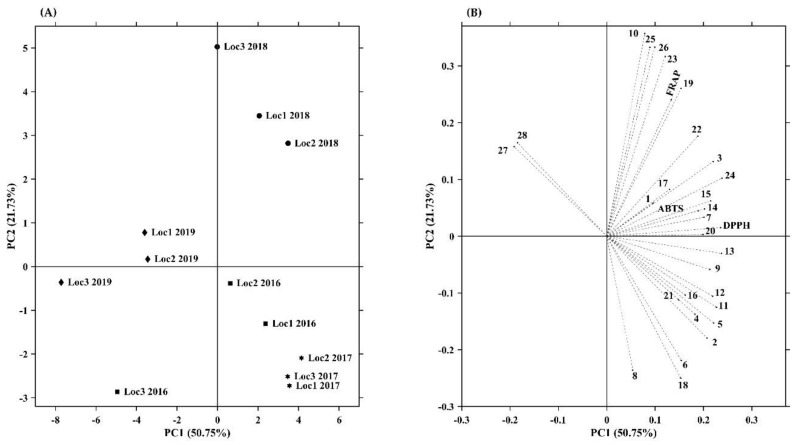
Principal component analysis (PCA) scores (**A**) and loadings plot (**B**) of phenolic composition and antioxidant capacity of *Prunus lusitanica* fruits. Abbreviations: Numbers 1–28 are phenolic compounds presented in Table 1 and Table 2; FRAP. ferric reducing antioxidant power; DPPH. scavenging capacity of DPPH radical; ABTS. scavenging capacity of ABTS radical; Loc. location.

Principal component analysis, PCA, is a very useful technique that allows the compression of information from many variables into a few uncorrelated variables, called principal components (PCs). PCA has been widely employed in multiple areas and fields, including discriminating the bioactive constituents and targeting them to certain bioactivity [20]. In this work, PCA was implemented using all samples (comprising different years and locations) to examine the inner relationships between the various phenolic compounds detected and the antioxidant capacity measured, allowing the identification of key patterns that most contribute to differentiating and characterizing samples. Thus, Figure 2 presents the scatter plot for the first two principal components, from which it is possible to analyze how samples cluster together in the reduced PCA subspace. (A) shows the scores plot and which variables contribute most to their separation, while (B) shows the loadings plot. This projection plan is defined by the two dimensions that best approximate the original data, i.e., the first two PCs, explaining altogether 72.5% (50.8% for PC1 and 21.7% for PC2) of the total variability present in the original dataset. The contribution of each variable to PC1 and PC2 is given in Table S3.

Regarding the two directions of PC1, two different paths can be observed (scores plot): four sets of samples follow a negative direction in PC1, while the other four sets of samples proceed in the positive direction, showing that they have roughly opposite responses. Interestingly, this fact seems to be mainly year-related, as 2019 samples move in one direction (negative side of PC1) and most of the remaining (2016, 2017, and 2018) samples follow the opposite direction (positive side of PC1). In addition, it can be verified that samples from 2018 are clustered in the upper right-hand (positive side of PC2) quadrant of the score plot, and samples from 2017 are located together in the lower right-hand corner (negative side of PC2). These remarks could indicate that environmental variables between years, such as temperature and precipitation, among others, have a considerable impact on the different fruit development stages and consequently on the phenolic composition and accumulation and subsequently the antioxidant capacity that is related to them [93,155].

The directions of the vectors regarding each variable (compound) in the loadings plot provide indicators about their potential importance and indicate correlations in the compounds’ variation patterns. The most important contributors to PC1 are compound **24** (0.238), **13** (0.237), DPPH (0.235), and compound **11** (0.227) (Table S3). Those are positioned close to each other, which indicates high positive correlations between them on the positive side and that they contribute similar information on PC1. Thus, samples from location 3 in 2019, location 1 in 2019, location 2 in 2019, and location 3 in 2016 are positioned on the left side of the score plot as the poorest source of phenolic compounds (except anthocyanins) and related antioxidant capacity. Moreover, the model interpretation suggests that samples from 2019 are characterized by higher levels of anthocyanins, which is in good agreement with the results presented in Table 2 and Figure 1. PC2 is positively correlated with compounds **10** (0.357), **25** (0.333), **26** (0.333), **23** (0.313), **19** (0.260), and FRAP (0.240) and negatively correlated with compounds **8**, **6**, and **18**. By analyzing the relationships between antioxidant capacity and phenolic composition, it can be concluded that anthocyanins are the less important phenolic compounds for the antioxidant capacities of samples located the farthest from them and that a large number of other individual phenolic compounds in cooperation strongly contribute to the antioxidant capacity, mainly the hydroxycinnamic acids and flavonols (which is in accordance with the results shown in Table S2).

## 4. Conclusions

The phenolic profile and content as well as the antioxidant capacity of *Prunus lusitanica* fruits were studied for the first time. Those proved to be an exceptional source of bioactive compounds regardless of the variations observed as a consequence of the different locations and years of study, showing high levels of hydroxycinnamic acids, flavan-3-ols, and anthocyanins. Among these compounds, significant correlations were found between the majority of the individual phenolic compounds within all identified classes and the antioxidant capacity measured by the DPPH, ABTS, and FRAP assays, allowing us to conclude that phenolic compounds are the main contributors to the high antioxidant capacity of *Prunus lusitanica* fruits. Through the obtained results, it can be stated that *Prunus lusitanica* fruits can be considered promising sources of natural bioactive compounds with antioxidant potential and are therefore suitable for future applications in the food and/or phytopharmaceutical industries. However, since this is the first study that characterizes and quantifies the phenolic compounds in *Prunus lusitanica* fruits as well as their antioxidant capacity, further studies approaching their composition and biological activities are needed.

## Figures and Tables

**Table 1:** Climatic conditions for the different seasons (2016, 2017, 2018, and 2019) in the district of Vila Real (https://tcktcktck.org/; accessed on 17 August 2022).

Season	Month																	
	January			February			March			April			May			June		
	Tm ^Z^	TM ^Y^	RR ^X^	Tm	TM	RR	Tm	TM	RR	Tm	TM	RR	Tm	TM	RR	Tm	TM	RR
**2016**	4.1	10.3	239.1	2.4	10.2	130.6	1.6	11.8	87.2	4.2	14.7	49.9	6.8	16.8	88.5	10.3	24.8	4.7
**2017**	0.0	9.6	41.0	3.4	11.6	126.7	3.4	14.1	41.0	5.1	19.6	12.1	8.6	21.7	78.4	12.1	27.7	15.5
**2018**	3.1	19.8	16.7	1.2	9.0	17.2	2.7	9.5	51.6	6.1	14.9	21.9	8.9	19.1	24.7	12.9	22.5	42.8
**2019**	3.7	10.5	40.7	5.3	14.2	14.9	6.2	16.7	48.2	7.1	16.0	61.7	11.1	22.8	12.5	12.9	23.0	18.1
	**July**			**August**			**September**			**October**			**November**			**December**		
	**Tm**	**TM**	**RR**	**Tm**	**TM**	**RR**	**Tm**	**TM**	**RR**	**Tm**	**TM**	**RR**	**Tm**	**TM**	**RR**	**Tm**	**TM**	**RR**
**2016**	13.5	29.7	14.8	12.8	30.6	13.5	10.7	26.1	11.0	8.1	18.3	60.7	3.9	12.3	116.9	2.4	11.3	49.5
**2017**	13.0	28.3	30.5	14.0	28.9	34.5	11.4	24.5	2.2	11.6	22.7	15.3	5.6	14.1	31.9	3.4	10.0	122.7
**2018**	14.1	25.0	15.0	15.9	30.4	1.9	15.5	27.7	19.4	9.0	18.4	13.7	6.2	11.5	44.6	5.3	11.2	17.9
**2019**	17.9	29.8	23.7	16.7	28.7	23.2	15.1	26.3	40.0	11.7	19.6	88.6	6.5	11.2	90.5	5.7	11.0	320.4

^Z^ Mean daily minimum air temperature (°C). ^Y^ Mean daily maximum air temperature (°C). ^X^ Total monthly rainfall (mm).

**Table 2:** (Poly)phenolic profile of *Prunus lusitanica* fruits.

Representative HPLC-DAD-ESI-MS/MS Chromatograms Showing the (poly)phenolic Profile Recorded at 280 and 520 nm					
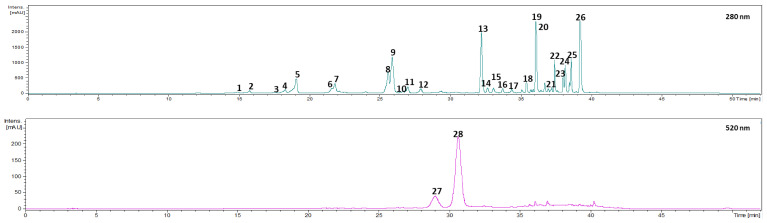					
Peak	Rt (min)	λ max (nm)	[M−H]^−^/[M+H]^+^ (*m*/*z*)	MS^n^ [M−H]^−^/[M+H]^+^ (%) (*m*/*z*)	Identification
**Hydroxicynamic acids**					
**1**	14.4	324	353/-	**MS^2^:** 191 (100.0), 179 (66.4)/-	3-*O*-Caffeoylquinic acid
				**MS^3^:** No fragments detected/-	
**2**	15.2	328	503/-	**MS^2^:** 161 (100.0), 341 (7.2), 323 (49.5), 179 (3.9)/-	Caffeoyl di-hexoside
				**MS^3^:** 143 (100.0), 135 (20.3)/-	
**3**	17.3	306	487/-	**MS^2^:** 145 (100.0), 341 (22.7), 307 (86.0), 173 (10.4), 163 (25.7)/-	*p*-coumaroyl-3-*O*-sucrose
				**MS^3^:** No fragments detected/-	
**4**	18.0	310	337/-	**MS^2^:** 163 (100.0), 191 (6.3)/-	3-*p*-coumaroylquinic acid
				**MS^3^:** 119 (100.0)/-	
**5**	18.9	312	487/-	**MS^2^:** 145 (100.0), 341 (6.9), 307 (89.8), 179 (1.6), 163 (22.6)/-	Caffeic acid-*O*-(coumaroyl)hexoside
				**MS^3^:** No fragments detected/-	
**7**	21.9	326	353/-	**MS^2^:** 173 (100.0), 191 (16.7), 179 (58.4), 135 (19.7)/-	Caffeoyl-isocitrate
				**MS^3^:** 111 (100.0), 155 (59.2), 127 (17.9)/-	
**10**	26.4	312	337/-	**MS^2^:** 173 (100.0), 163 (7.4)/-	4-*p*-coumaroylquinic acid
				**MS^3^:** 137 (100.0), 155 (14.2), 127 (8.3)/-	
**11**	27.0	312	529/-	**MS^2^:** 487 (100.0), 349 (13.4), 307 (75.9), 162 (29.0), 173 (2.4)/-	mono-*O*-acetyl-3-*O*-*p*-coumaroylsucrose isomer
				**MS^3^:** 145 (100.0), 307 (43.7)/-	
**12**	27.4	314	529/-	**MS^2^:** 487 (100.0), 349 (13.4), 307 (69.0), 173 (2.4), 162 (29.0)/-	mono-*O*-acetyl-3-*O*-*p*-coumaroylsucrose isomer
				**MS^3^:** 145 (100.0), 307 (43.7)/-	
**13**	32.4	314	571/-	**MS^2^:** 529 (100.0), 511 (51.7), 393 (10.2), 383 (10.1), 341 (7.8)/-	di-*O*-acetyl-3-*O*-*p*-coumaroyl sucrose isomer
				**MS^3^:** 349 (100.0), 487 (49.6), 469 (20.1), 341 (17.6), 307 (89.6)/-	
**14**	32.7	308	571/-	**MS^2^:** 529 (100.0), 511 (56.3), 469 (4.3), 425 (7.1), 383 (14.7), 349 (26.5), 307 (77.1), 217 (14.1), 173 (3.4)/-	di-*O*-acetyl-3-*O*-*p*-coumaroyl sucrose isomer
				**MS^3^:** 487 (100.0), 469 (10.9), 367 (33.3), 341 (8.1), 307 (62.3), 217 (5.7), 171 (11.9), 163 (14.7)/-	
**15**	33.2	314	571/-	**MS^2^:** 529 (100.0), 511 (26.6), 425 (6.2), 383 (8.0), 341 (4.6), 307 (63.1)/-	di-*O*-acetyl-3-*O*-*p*-coumaroyl sucrose isomer
				**MS^3^:** 487 (100.0), 469 (20.2), 383 (18.2), 367 (4.5), 341 (25.8), 307 (9.7), 290 (13.1)/-	
**16**	33.8	314	613/-	**MS^2^:** 571 (100.0), 553 (18.0), 449 (4.0), 425 (5.7), 349 (2.6)/-	tri-*O*-acetyl-3-*O*-*p*-coumaroyl sucrose isomer
				**MS^3^:** 425 (100.0), 559 (10.8), 451 (12.5), 407 (68.1), 289 (13.6), 273 (8.1)/-	
**17**	34.9	308	613/-	**MS^2^:** 571 (100.0), 553 (34.5), 425 (2.4), 349 (16.9), 217 (4.2)/-	tri-*O*-acetyl-3-*O*-*p*-coumaroyl sucrose isomer
				**MS^3^:** 529 (100.0), 511 (58.7), 425 (23.7), 383 (38.2), 349 (92.1), 289 (11.1), 217 (13.3), 163 (26.2)/-	
**18**	35.9	306	571/-	**MS^2^:** 511 (100.0), 529 (22.2), 487 (2.3), 469 (4.7), 451 (9.8), 349 (7.3), 307 (11.5), 289 (4.3), 259 (3.4), 214 (3.9)/-	di-*O*-acetyl-3-*O*-*p*-coumaroyl sucrose isomer
				**MS^3^:** 451 (100.0), 469 (80.2), 422 (8.7), 349 (4.0), 331 (24.9), 289 (31.4), 271 (22.5), 260 (22.5), 231 (11.1), 214 (68.3), 173 (33.9), 145 (66.5)/-	
**19**	36.2	310	613/-	**MS^2^:** 571 (100.0), 553 (40.9), 529 (5.9), 467 (4.1), 425 (12.8), 383 (6.0), 349 (23.1), 228 (3.9), 201 (8.4)/-	tri-*O*-acetyl-3-*O*-*p*-coumaroyl sucrose isomer
				**MS^3^:** 349 (100.0), 529 (82.5), 511 (46.5), 487 (4.1), 469 (5.3), 425 (16.8), 407 (6.6), 383 (26.9), 307 (13.5), 277 (11.0), 219 (9.6), 163 (10,6)/-	
**22**	37.6	316	613,5/-	**MS^2^:** 553 (100.0), 571 (31.3), 511 (6.7), 425 (4.3), 349 (28.9), 289 (4.5)/-	tri-*O*-acetyl-3-*O*-*p*-coumaroyl sucrose isomer
				**MS^3^:** 493 (100.0), 511 (64.6), 469 (9.8), 365 (6.9), 349 (27.9), 331 (10.2), 307 (4.5), 271 (5.9), 269 (32.8), 245 (10.1), 214 (49.4), 187 (10.2), 163 (9.8)/-	
**23**	38.1	316	613/-	**MS^2^:** 553 (100.0), 571 (34.3), 511 (5.9), 493 (10.3), 425 (7.2), 407 (7.1), 391 (6.9), 331 (5.9)/-	tri-*O*-acetyl-3-*O*-*p*-coumaroyl sucrose isomer
				**MS^3^:** 493 (100.0), 511 (68.9), 469 (4.4), 451 (15.0), 391 (6.6), 303 (6.7), 287 (30.5), 271 (4.7), 214 (20.7), 197 (12.2)/-	
**24**	38.2	318	613/-	**MS^2^:** 553 (100.0), 571 (39.7), 511 (4.2), 494 (18.1), 407 (3.7), 391 (11.7), 349 (4.1), 318 (5.6)/-	tri-*O*-acetyl-3-*O*-*p*-coumaroyl sucrose isomer
				**MS^3^:** 493 (100.0), 511 (86.1), 451 (5.7), 389 (3.3), 349 (4.6), 331 (22.4), 245 (22.5), 163 (19.2)/-	
**25**	38.6	316	655/-	**MS^2^:** 595 (100.0), 613 (44.9), 553 (16.7), 535 (14.1), 494 (7.5), 391 (23.2), 349 (18.4), 330 (11.5), 313 (10.0), 270 (4.5)/-	tetra-*O*-acetyl-3-*O*-*p*-coumaroylsucrose isomer
				**MS^3^:** 535 (100.0), 553 (44.9), 493 (41.3), 331 (19.5), 287 (17.1)/-	
**26**	39.3	398, sh 334	655/-	**MS^2^:** 595 (100.0), 613 (20.1), 553 (4.5), 535 (26.7), 393 (14.7), 331 (5.0)/-	tetra-*O*-acetyl-3-*O*-*p*-coumaroylsucrose isomer
				**MS^3^:** 553 (100.0), 535 (77.7), 511 (5.5), 493 (19.5)/-	
**Secoiridoids**					
**6**	21.6	328	581/-	**MS^2^:** 545 (100.0), 503 (1.8)/-	6’-*O*-*β*-D-glucosyl swertiamarin (tentative)
				**MS^3^:** 503 (42.6), 341 (28.8), 323 (52.2), 235 (39.1), 161 (100.0)/-	
**Flavan-3-ols**					
**8**	25.3	278	289/-	**MS^2^:** 245 (100.0), 205 (38.1), 179 (11.6), 165 (2.9)/-	Catechin
				**MS^3^:** 203 (100.0)/-	
**9**	25.9	280	865/-	**MS^2^:** 695 (100.0), 739 (44.3), 713 (32.1), 577 (53.3), 575 (26.5), 451 (6.9), 407 (23.4), 363 (5.9), 289 (16.9), 287 (8.7)/-	B-type proanthocyanidin trimer
				**MS^3^:** 173 (100.0), 163 (6.4)/-	
**Flavonols**					
**20**	36.9	340	463/-	**MS^2^:** 301 (100.0), 271 (4.7), 179 (6.8)/-	Quercetin-3-*O*-glucoside
				**MS^3^:** 179 (100.0), 271 (31.4), 255 (31.8), 229 (5.9), 213 (13.2), 193 (7.9), 151 (86.4), 121 (7.9)/-	
**21**	37.1	356	609/-	**MS^2^:** 301 (100.0), 179 (4.5)/-	Quercetin-3-*O*-rutinoside
				**MS^3^:** 179 (100.0), 271 (57.7), 255 (21.4), 229 (11.1), 211 (3.7), 193 (3.5), 151 (75.1), 121 (2.9), 107 (5.2)/-	
**Anthocyanins**					
**27**	28.6	518	-/449	**MS^2^:** -/287 (100.0), 366 (1.0), 307 (0.7)	Cyanidin-3-*O*-glucoside
				**MS^3^:** -/227 (44.0), 213 (25.3), 203 (10.9), 187 (100.0), 160 (41.2)	
**28**	30.4	520	-/595	**MS^2^:** -/287 (100.0), 467 (5.5), 329 (7.8)	Cyanidin-3-(6-*trans*-*p*-coumaroyl)glucoside
				**MS^3^:** -/259 (100.0), 269 (14.2), 219 (54.7), 127 (56.5)	

Retention time (Rt), wavelengths of maximum absorption in the UV–vis region (λ max), pseudomolecular and MS^n^ fragment ions.

**Table 3:** Quantitative (poly)phenolic profile (mg/100g fw) of *Prunus lusitanica* fruits grown under different agroclimatic conditions (locations and years).

Peak	Location and Year																		
	Location 1					Location 2					Location 3								
	2016	2017	2018	2019	LSD	2016	2017	2018	2019	LSD	2016	2017	2018	2019	LSD	LSD (*p* < 0.05) for Location Comparison			
					(*p* < 0.05)					(*p* < 0.05)					(*p* < 0.05)	2016	2017	2018	2019
**Phenolic acids**																			
**1**	2.34 B c	1.48 A a	1.65 A a	2.18 B ab	0.25	1.84 A b	2.68 B b	4.05 C b	2.92 B b	0.52	1.35 A a	3.02 C b	1.90 B a	1.74 AB a	0.31	0.23	0.44	0.47	0.42
**2**	20.26 B b	28.96 C a	17.18 B b	3.96 A b	2.33	22.30 B b	24.91 B a	22.80 B b	5.55 A c	2.84	14.94 B a	28.01 C a	4.41 A a	2.68 A a	2.28	2.47	2.93	3.57	0.76
**3**	21.64 C b	19.27 B a	23.10 D a	11.60 A b	0.90	15.90 B a	19.45 C a	22.74 D a	11.83 A b	0.92	13.02 B a	18.10 C a	21.27 D a	8.21 A a	1.89	0.79	1.03	1.45	0.93
**4**	0.11 C c	0.11 C c	0.05 B a	0.03 A c	0.01	0.08 C b	0.08 C b	0.06 B a	0.02 A b	0.01	0.04 B a	0.05 C a	0.04 B a	0.01 A a	0.01	0.01	0.01	0.01	0.01
**5**	0.73 C c	0.99 D a	0.50 B a	0.33 A b	0.06	0.62 B b	0.97 D a	0.79 C b	0.46 A c	0.04	0.47 B a	1.09 C b	0.51 B a	0.16 A a	0.03	0.07	0.03	0.05	0.03
**7**	16.49 B b	13.64 A a	14.27 A a	12.75 A c	1.46	16.14 B b	16.68 B b	11.88 A a	10.84 A b	1.26	7.11 A a	16.14 B b	15.37 B a	7.81 A a	2.66	1.85	1.80	2.85	1.17
**10**	103.26 A b	96.47 A a	169.42 C a	122.38 B ab	5.98	104.75 A b	109.69 A a	161.80 C a	124.62 B b	6.57	72.31 A a	111.77 C a	177.24 D a	95.64 B a	9.68	5.37	6.43	8.40	10.88
**11**	28.11 BC b	32.63 C a	23.86 B b	18.39 A b	3.69	30.99 C b	31.21 C a	24.93 B b	19.82 A b	2.75	17.27 B a	32.14 D a	19.69 C a	9.61 A a	0.52	5.23	0.45	0.72	1.58
**12**	14.49 BC b	15.07 C a	14.10 B b	6.91 A b	0.69	10.72 B a	14.20 C a	14.07 C b	5.12 A a	1.48	9.27 B a	14.30 C a	6.46 A a	5.40 A a	0.90	1.66	0.39	0.71	0.61
**13**	257.14 BC cb	263.34 C a	238.79 B b	155.65 A b	56.04	181.51 A a	271.56 B a	284.75 B c	190.54 A c	15.24	160.10 B a	300.28 D a	204.93 C a	109.72 A a	12.85	13.11	120.75	12.60	9.05
**14**	12.19 B ab	13.75 C ab	12.49 B b	9.52 A b	1.28	12.32 B b	13.76 C b	12.07 B b	8.63 A ab	0.85	4.48 A a	7.88 C a	9.26 D a	5.57 B a	0.62	0.53	0.06	0.82	1.71
**15**	16.47 B b	23.05 C ba	17.19 B a	13.18 A b	0.92	13.71 A a	22.56 C a	21.19 C b	15.93 B b	1.04	10.33 A a	21.28 B a	22.74 B b	8.23 A a	1.66	1.74	0.29	1.51	0.74
**16**	12.55 D b	10.74 C a	8.11 B b	5.34 A b	1.11	15.72 D c	10.20 C a	6.84 B b	4.83 A ab	1.19	4.39 A a	8.59 B a	5.64 A a	3.92 A a	1.58	0.66	1.40	1.11	0.70
**17**	28.30 A a	37.78 A a	87.94 C b	70.12 B b	10.00	33.68 B a	60.46 C b	95.38 D b	15.15 A a	7.10	35.84 C a	56.05 D ab	14.40 B a	6.53 A a	4.06	6.02	17.15	10.80	5.41
**18**	17.74 D b	13.70 C b	5.47 B c	3.04 A a	0.58	5.95 B a	17.00 C c	3.51 A b	5.47 B b	1.03	4.73 B a	11.54 C a	N.d.	2.41 A a	1.01	1.34	0.31	0.42	0.63
**19**	352.54 C b	234.09 A a	482.67 D b	275.78 B b	10.38	364.38 B b	309.66 A a	461.57 C b	296.34 A b	32.49	163.69 A a	312.07 C a	366.67 D a	189.65 B a	9.49	36.35	76.83	13.39	8.63
**22**	87.37 C b	74.79 B a	97.76 D a	43.14 A b	5.23	83.22 B b	78.52 B a	107.10 C b	22.41 A a	3.40	44.76 A a	80.13 B a	108.56 C b	44.62 A b	1.27	3.88	6.37	4.03	2.03
**23**	43.30 B b	36.83 A a	52.98 C a	38.68 AB b	4.55	40.16 A b	39.54 A a	49.43 B a	41.59 A b	4.91	19.02 A a	35.87 C a	64.19 D b	27.02 B a	3.46	5.86	8.93	3.59	1.56
**24**	71.58 B b	71.91 B a	74.26 B a	46.21 A b	6.56	59.96 B ab	73.10 C a	84.70 D b	49.09 A b	7.79	42.92 B a	66.63 C a	68.96 C a	32.60 A a	4.15	9.87	16.43	3.40	2.60
**25**	43.45 A b	48.56 A a	66.55 B a	51.30 A b	10.54	52.62 B b	37.18 A a	60.45 C a	42.36 A a	5.54	27.50 A a	41.44 B a	65.15 C a	39.60 B a	4.96	6.40	11.01	8.50	3.94
**26**	1008.55 B a	598.98 A a	1313.44 C a	712.29 A a	100.40	1025.47 B a	750.80 A b	1225.35 C a	810.41 A a	123.11	421.65 A a	774.57 B b	1462.26 C a	662.15 B a	138.66	144.34	81.59	173.37	95.95
**Secoiridoids**																			
**6**	9.89 B b	10.99 B a	8.26 A b	7.38 A b	0.85	9.29 C ab	11.23 D b	4.99 A a	8.04 B b	0.61	7.11 B a	14.98 C c	6.29 B ab	4.23 A a	0.80	0.95	0.03	1.16	0.49
**Flavan-3-ols**																			
**8**	15.76 A a	19.78 B a	19.78 B c	20.95 B a	1.82	14.46 AB a	20.08 C a	11.53 A b	17.90 BC a	3.16	14.34 A a	30.57 B b	N.d.	14.59 A a	1.97	0.93	3.64	1.44	2.78
**9**	1157.98 B c	902.07 A a	830.41 A b	784.92 A b	85.31	965.50 B b	1096.20 B b	1302.16 C c	675.23 A b	94.19	743.17 B a	1159.16 C b	669.99 B a	338.88 A a	86.78	71.13	89.18	117.69	93.21
**Flavonols**																			
**20**	2.84 A b	5.10 B b	2.45 A a	2.83 A b	0.44	2.78 A b	5.14 B b	6.31 B b	2.14 A b	0.95	1.42 B a	3.16 D a	2.64 C a	0.75 A a	0.29	0.17	1.13	0.65	0.18
**21**	30.27 B b	41.70 C a	25.36 A a	29.99 B b	2.96	14.96 A a	37.46 D a	33.89 C b	22.30 B b	1.83	34.20 C b	36.68 C a	29.72 B ab	13.40 A a	3.13	2.74	3.93	2.36	2.11
**Anthocyanins**																			
**27**	141.29 B a	41.03 A a	214.93 C b	450.54 D b	17.21	133.45 B a	57.44 A b	153.98 C a	268.65 D a	10.42	224.93 B b	44.14 A a	225.52 B b	284.24 C a	25.03	13.26	5.46	32.08	17.73
**28**	107.17 B a	31.35 A a	166.41 C b	369.70 D c	9.55	119.74 B b	34.71 A a	134.40 C a	248.87 D b	6.68	191.41 B c	35.64 A a	194.69 B b	200.85 B a	10.24	6.75	3.39	7.71	15.65

In the same row, different capital letters mean significant differences between years for each location according to the one-way ANOVA and Tukey’s multiple range test at *p* < 0.05. In the same row, values with different lowercase letters are significantly different between locations in the same year according to the one-way ANOVA and Tukey’s multiple range test at *p* < 0.05. N.d., not detected.

**Table 4:** In vitro antioxidant capacity (mmol TE/100g fw) of *Prunus lusitanica* fruits grown under different agroclimatic conditions (locations and years).

Peak	Location and Year																		
	Location 1					Location 2					Location 3					LSD (*p* < 0.05) for Location Comparison			
	2016	2017	2018	2019	LSD (*p* < 0.05)	2016	2017	2018	2019	LSD (*p* < 0.05)	2016	2017	2018	2019	LSD (*p* < 0.05)	2016	2017	2018	2019
**ABTS^•+^**	9.20 A b	10.49 C ab	10.45 C b	9.94 B b	0.28	9.46 A b	10.69 C b	9.61 AB a	9.91 B b	0.26	8.23 A a	10.32 C a	9.80 B a	7.88 A a	0.28	0.32	0.24	0.28	0.32
**DPPH^•^**	7.19 B b	7.69 C a	7.35 B a	6.78 A b	0.20	7.255 B b	8.17 C b	7.49 B a	6.67 A b	0.17	6.18 B a	8.04 D b	7.56 C a	5.18 A a	0.10	0.20	0.20	0.22	0.10
**FRAP**	9.22 A a	9.99 B a	11.76 C b	8.99 A a	0.49	9.209 A a	9.98 B a	10.26 B a	9.04 A a	0.44	8.76 A a	10.25 B a	11.26 C b	9.26 A a	0.47	0.49	0.47	0.49	0.49

In the same row, different capital letters mean significant differences between years for each location according to the one-way ANOVA and Tukey’s multiple range test at *p* < 0.05. In the same row, values with different lowercase letters are significantly different between locations in the same year according to the one-way ANOVA and Tukey’s multiple range test at *p* < 0.05. N.d. not detected.

## Data Availability

All data are contained in this article and Supplementary Materials.

## Supplementary Materials

The following supporting information can be downloaded at: https://www.mdpi.com/article/10.3390/antiox11091738/s1, Table S1: Fruit water content (expressed in %); **Table S2**: Correlation matrix—Pearson’s correlation factor values (*r*); Table S3: Contribution of the different phenolic compounds and antioxidant methods to the PCA factors (PC1 and PC2).
