# Supplementary material for: Prunus lusitanica L. Fruits as a Novel Source of Bioactive Compounds with Antioxidant Potential: Exploring the Unknown
Source: Antioxidants (Basel). 2022 Aug 31;11(9):1738. doi: 10.3390/antiox11091738 (PMC9495831; doi:10.3390/antiox11091738)
Supplement: Supplementary file 1 [file antioxidants-11-01738-s001.zip › antioxidants-1844250-supplementary.pdf]

ARTICLE

PRUNUS LUSITANICA L. FRUITS AS A NOVEL SOURCE OF BIOACTIVE COMPOUNDS WITH ANTIOXIDANT POTENTIAL: EXPLORING THE UNKNOWN

Table S1. Fruits water content (expressed in %)

	Year of Study			
**Location**	**2016**	**2017**	**2018**	**2019**
**BLOC**	62.5	55.9	60.1	68.9
**BIB**	63.3	55.7	62.1	66.5
**P2**	70.0	56.2	63.0	69.7

Table S2. Correlation matrix – Pearson's correlation factor values (*r*)

	1	2	3	4	5	7	10	11	12	13	14	15	16	17	18	19	22	23	24	25	26	6	8	9	20	21	27	28	ABTS	DPPH	FRAP
**1**	1,000																														
**2**	0,212	1,000																													
**3**	0,260	0.583*	1,000																												
**4**	-0,056	0.763**	0.591*	1,000																											
**5**	0,377	0.902***	0.612*	0.675*	1,000																										
**7**	0,189	0,504	0.659*	0.603*	0.621*	1,000																									
**10**	0,343	-0,196	0,565	-0,210	-0,065	0,306	1,000																								
**11**	0,230	0.894***	0.626*	0.799**	0.905***	0.786**	-0,037	1,000																							
**12**	0,210	0.925***	0.748**	0.772**	0.8120**	0,547	-0,003	0.814**	1,000																						
**13**	0,533	0.798**	0.813**	0.590*	0.908***	0.647*	0,272	0.813**	0.849**	1,000																					
**14**	0,192	0,568	0.690*	0.757**	0,541	0.725**	0,314	0.733**	0.657*	0.619*	1,000																				
**15**	0,404	0,544	0.763**	0,460	0.784**	0.662*	0,470	0.685*	0,564	0.848**	0.6230*	1,000																			
**16**	-0,046	0.691*	0,449	0.840**	0,549	0.735**	-0,164	0.814**	0.625*	0,436	0.709*	0,286	1,000																		
**17**	0,422	0,419	0,494	0,118	0,325	0,218	0,401	0,330	0.577*	0,513	0,433	0,317	0,080	1,000																	
**18**	0,090	0.703*	0,351	0.780**	0.725**	0,533	-0,419	0.720**	0.708*	0.619*	0,515	0,391	0.613*	0,055	1,000																
**19**	0,450	0,231	0.757**	0,191	0,211	0,557	0.811**	0,356	0,409	0,524	0.603*	0,462	0,325	0.586*	-0,048	1,000															
**22**	0,191	0,476	0.898***	0,467	0,466	0.629*	0.591*	0,495	0.609*	0.626*	0,569	0.636*	0,450	0,407	0,127	0.737**	1,000														
**23**	0,265	-0,071	0.682*	0,098	0,097	0.578*	0.913***	0,195	0,096	0,373	0,522	0.607*	0,137	0,220	-0,167	0.809**	0.686*	1,000													
**24**	0,430	0.671*	0.961***	0.626*	0.724**	0.690*	0,531	0.730**	0.785**	0.891***	0.784**	0.845*	0,497	0,555	0,410	0.753**	0.847**	0.650*	1,000												
**25**	0,114	-0,077	0,567	0,020	-0,064	0,384	0.888***	0,091	0,095	0,206	0,477	0,395	0,112	0,384	-0,390	0.800**	0.659*	0.872***	0,537	1,000											
**26**	0,250	-0,072	0.668*	0,035	-0,019	0,478	0.894***	0,101	0,102	0,283	0,403	0,406	0,164	0,238	-0,278	0.879***	0.756**	0.929***	0.597*	0.872***	1,000										
**6**	0,072	0.695*	0,279	0,495	0.788**	0.642*	-0,278	0.803**	0.592*	0.631*	0,317	0,473	0,546	0,113	0.739**	-0,005	0,118	-0,094	0,341	-0,249	-0,221	1,000									
**8**	0,145	0,449	-0,139	0,107	0,414	0,151	-0,396	0,400	0,406	0,321	0,064	0,012	0,175	0,343	0,507	-0,147	-0,306	-0,449	-0,036	-0,380	-0,512	0.705*	1,000								
**9**	0.607*	0.796**	0.691*	0.632*	0.802**	0.609*	0,111	0.799**	0.814**	0.855***	0.597*	0.59*	0.576*	0.607*	0,570	0,522	0,562	0,218	0.789**	0,112	0,212	0,524	0,293	1,000							
**20**	0,567	0.656*	0.623*	0,559	0.729**	0,440	0,257	0.660*	0.662*	0.762**	0.730**	0.770**	0,356	0.586*	0,396	0,417	0,520	0,323	0.794**	0,272	0,186	0,254	0,084	0.756**	1,000						
**21**	0,199	0,559	0,488	0,426	0.724**	0,250	-0,028	0,506	0.590*	0.690*	0,276	0.677*	0,031	0,397	0,493	-0,037	0,264	0,020	0,529	-0,118	-0,183	0,484	0,251	0,559	0.638*	1,000					
**27**	-0,168	(-0.883)***	-0,566	(-0.690)*	(-0.878)***	-0,501	0,155	(-0.804)**	(-0.768)**	(-0.754)**	-0,461	(-0.624)*	(-0.617)*	-0,072	(-0.681)*	-0,154	-0,501	-0,022	(-0.629)*	0,118	-0,015	(-0.654)*	-0,233	(-0.604)*	-0,534	-0,456	1,000				
**28**	-0,120	(-0.871)***	-0,545	(-0.689)*	(-0.845)**	-0,485	0,185	(-0.764)**	(-0.783)**	(-0.724)**	-0,451	(-0.579)*	(-0.607)*	-0,075	(-0.704)*	-0,123	-0,505	0,017	(-0.598)*	0,137	0,011	(-0.634)*	-0,256	-0,564	-0,504	-0,432	0.988***	1,000			
**ABTS**	0,276	0,451	0,560	0,355	0.625*	0.710*	0,427	0.693*	0,488	0.692*	0.707*	0.797**	0,339	0,485	0,385	0,487	0,358	0,526	0.664*	0,408	0,285	0.600*	0,363	0,489	0.613*	0,502	-0,369	-0,325	1,000		
**DPPH**	0,360	0.689*	0.776**	0,568	0.845**	0.837**	0,358	0.866***	0.691*	0.870***	0.708*	0.899***	0,536	0,462	0,503	0,538	0.645*	0,525	0.852***	0,340	0,370	0.671*	0,204	0.761**	0.729**	0.631*	(-0.646)*	(-0.594)*	0.861***	1,000	
**FRAP**	0,045	0,173	0.724**	0,040	0,244	0,422	0.790**	0,219	0,351	0,479	0,374	0.619*	0,018	0,394	-0,103	0.671*	0.743**	0.739**	0.645*	0.734**	0.725**	0,076	-0,163	0,148	0,275	0,203	-0,269	-0,279	0,529	0,523	1,000

Statistically significant correlations: *$p < 0.05$ **$p < 0.01$ ***$p < 0.001$

Abbreviations: Numbers 1–28 are phenolic compounds presented in Tables 1 and 2;

ABTS, scavenging capacity of ABTS radical; FRAP, ferric reducing antioxidant power; DPPH, scavenging capacity of DPPH radical

Table S3. Contribution of the different phenolic compounds and antioxidant methods to the PCA factors (PC1 and PC2).

Loadings	PC1	PC2
**1**	0,096	0,058
**2**	0,207	-0,179
**3**	0,220	0,131
**4**	0,182	-0,138
**5**	0,221	-0,153
**7**	0,201	0,033
**10**	0,079	0,357
**11**	0,227	-0,125
**12**	0,219	-0,106
**13**	0,237	-0,030
**14**	0,202	0,048
**15**	0,215	0,062
**16**	0,162	-0,104
**17**	0,130	0,082
**18**	0,153	-0,250
**19**	0,154	0,260
**22**	0,188	0,176
**23**	0,121	0,316
**24**	0,238	0,102
**25**	0,089	0,333
**26**	0,099	0,333
**6**	0,154	-0,219
**8**	0,054	-0,236
**9**	0,213	-0,059
**20**	0,199	0,003
**21**	0,148	-0,112
**27**	-0,191	0,158
**28**	-0,185	0,165
**ABTS**	0,189	0,045
**DPPH**	0,235	0,015
**FRAP**	0,133	0,240

.
